# Frequent *LPA* KIV-2 Variants Lower Lipoprotein(a) Concentrations and Protect Against Coronary Artery Disease

**DOI:** 10.1016/j.jacc.2021.05.037

**Published:** 2021-08-03

**Authors:** Johanna F. Schachtl-Riess, Azin Kheirkhah, Rebecca Grüneis, Silvia Di Maio, Sebastian Schoenherr, Gertraud Streiter, Jamie Lee Losso, Bernhard Paulweber, Kai-Uwe Eckardt, Anna Köttgen, Claudia Lamina, Florian Kronenberg, Stefan Coassin, for the GCKD Investigators

**Affiliations:** a Institute of Genetic Epidemiology, Department of Genetics and Pharmacology, Medical University of Innsbruck, Innsbruck, Austria; b First Department of Internal Medicine, Paracelsus Private Medical University, Salzburg, Austria; c Department of Nephrology and Hypertension, Friedrich-Alexander Universität Erlangen-Nürnberg, Erlangen, Germany; d Department of Nephrology and Medical Intensive Care, Charité—Universitätsmedizin Berlin, Berlin, Germany; e Institute of Genetic Epidemiology, Faculty of Medicine and Medical Center, University of Freiburg, Freiburg, Germany

**Keywords:** cardiovascular disease, cohort study, copy number variation, genetic variability, lipoprotein(a), Mendelian randomization

## Abstract

**Background:**

Lipoprotein(a) (Lp(a)) concentrations are a major independent risk factor for coronary artery disease (CAD) and are mainly determined by variation in *LPA*. Up to 70% of the LPA coding sequence is located in the hyper-variable kringle IV type 2 (KIV-2) region. It is hardly accessible by conventional technologies, but may contain functional variants.

**Objectives:**

This study sought to investigate the new, very frequent splicing variant KIV-2 4733G>A on Lp(a) and CAD.

**Methods:**

We genotyped 4733G>A in the GCKD (German Chronic Kidney Disease) study (n = 4,673) by allele-specific polymerase chain reaction, performed minigene assays, identified proxy single nucleotide polymorphisms and used them to characterize its effect on CAD by survival analysis in UK Biobank (n = 440,234). Frequencies in ethnic groups were assessed in the 1000 Genomes Project.

**Results:**

The 4733G>A variant (38.2% carrier frequency) was found in most isoform sizes. It reduces allelic expression without abolishing protein production, lowers Lp(a) by 13.6 mg/dL (95% CI: 12.5-14.7; P < 0.0001) and is the strongest variance-explaining factor after the smaller isoform. Splicing of minigenes was modified. Compound heterozygosity (4.6% of the population) for 4733G>A and 4925G>A, another KIV-2 splicing mutation, reduces Lp(a) by 31.8 mg/dL and most importantly narrows the interquartile range by 9-fold (from 42.1 to 4.6 mg/dL) when compared to the wild type. In UK Biobank 4733G>A alone and compound heterozygosity with 4925G>A reduced HR for CAD by 9% (95% CI: 7%-11%) and 12% (95% CI: 7%-16%) (both P < 0.001). Frequencies in ethnicities differ notably.

**Conclusions:**

Functional variants in the previously inaccessible *LPA* KIV-2 region cooperate in determining Lp(a) variance and CAD risk. Even a moderate but lifelong genetic Lp(a) reduction translates to a noticeable CAD risk reduction. (J Am Coll Cardiol 2021;78:437–49)

## Graphical abstract

**Graphical abstract:**
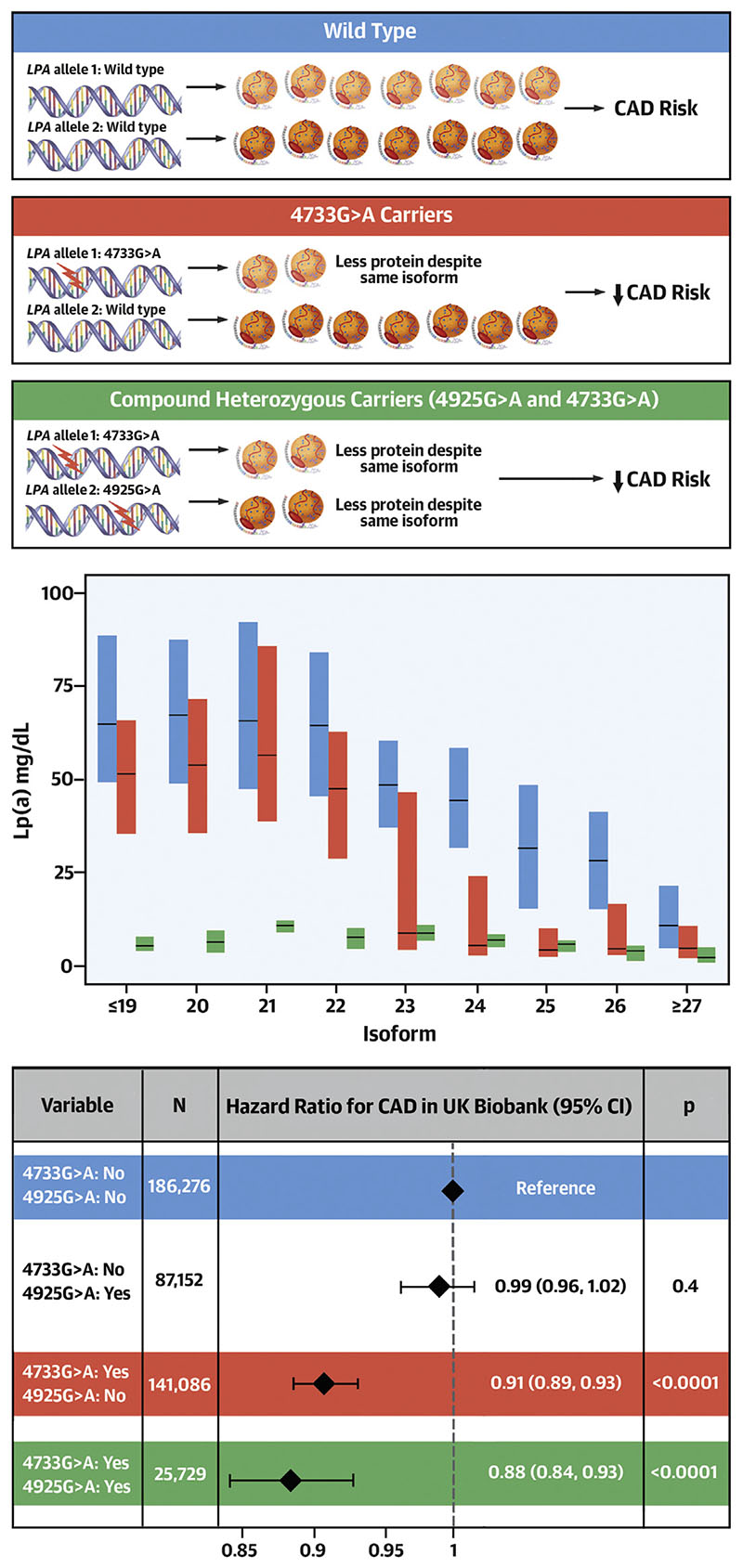
CENTRAL ILLUSTRATION Frequent *LPA* KIV-2 Variants Lower Lipoprotein(a) and Coronary Artery Disease Risk The frequent *LPA* kringle IV type 2 (KIV-2) variant 4733G>A and compound heterozygosity for 4733G>A and 4925G>A are associated with lower lipoprotein(a) (Lp(a)) concentrations and a lower risk for coronary artery disease (CAD).

The lipoprotein(a) (Lp(a)) trait is a major riddle. Lp(a) has several proin-flammatory, proatherogenic and potentially also prothrombotic properties (1) and has been associated with cardiovascular diseases, including coronary artery disease (CAD), heart failure, aortic valve stenosis, peripheral artery disease, stroke, and cardiovascular and total mortality (2,3). Although recent findings point toward oxidized phospholipids as a major determinant of Lp(a) pathogenicity inducing inflammation of the vascular wall (4–6), many details of both the pathophysiology and especially the genetic regulation of Lp(a) are still unclear.

Individual Lp(a) concentrations present a 1,000-fold concentration range in the general population, and median concentrations differ by nearly 10-fold between ethnicities (7) and 2- to 3-fold even within Europe (8). Unlike other lipoproteins, which show a highly polygenic genetic architecture, >90% of the Lp(a) variance is controlled by 1 gene, LPA, which encodes apolipoprotein(a) (apo(a)) (9). The gene structure of LPA is complex, and most of the coding sequence is not accessible for conventional sequencing or genotyping technologies. LPA has a highly repetitive structure consisting of 10 highly homologous kringle IV (KIV) domains (subtypes 1 to 10), a kringle V domain and a protease domain (9). The kringle IV type 2 (KIV-2) domain is encoded in a 5.6-kilobase large DNA copy number variation that can be present up to >40× per allele and creates >40 protein isoforms (9). The isoform size is inversely (but not linearly) correlated with the Lp(a) concentrations with carriers of low molecular weight (LMW) isoforms (11-22 KIV repeats) presenting 5× to 10 × higher median Lp(a) concentrations than carriers of high molecular weight isoforms (> 22 repeats)(9). Interestingly, whereas 1 LMW allele is commonly sufficient to cause increased Lp(a) concentrations, the Lp(a) concentrations of 2 individuals with the same isoform combination can still differ 200-fold (10). Indeed, in nearly every isoform group individuals with either very high or very low Lp(a) values are found. This means that besides the KIV size polymorphism other genetic variants exist that have a strong influence on Lp(a) concentrations.

We have recently performed a comprehensive mutation screening of the KIV-2 region by deep sequencing in individuals with a discrepancy between observed and expected Lp(a) values based on the apo(a) isoform size (11). This revealed the splice site variant LPA KIV-2 4925G>A with a 22% carrier frequency and a high impact on Lp(a) concentrations. It occurs specifically in LMW isoforms, reduces Lp(a) by 30 mg/dL, and decreases protein expression without leading to a null allele (11). It explained some part of several peculiar aspects of the Lp(a) trait (9) and exemplified how investigation of variants located in the poorly covered KIV-2 region can provide novel insights into the genetics of the Lp(a) trait (11).

In the same sequencing data set (11), we noted a second variant, named here *LPA* KIV-2 4733G>A. It was located only 11 base pairs (bp) before the splice acceptor of the second exon of a KIV-2 repeat and was seen in 49 of 123 samples.The variant is located in the potentially functional region preceding the splice site acceptor and preliminary analyses in those 43 carriers had indicated an effect on Lp(a) (12). In the present study we investigate this variant and its functional consequences on Lp(a) concentrations and CAD risk in several large cohorts. We found that it is the second strongest genetic contributor to Lp(a) variance besides the KIV size polymorphism and shows a protective effect on cardiovascular outcomes in UK Biobank.

## Methods

### Variant Typing

The nonstandard naming of the variant is caused by the complexity of the KIV-2 region (rationale detailed in the Supplemental Methods). Possible genome coordinates are given in Supplemental Table 1. The 4733G>A variant was typed using an allele-specific polymerase chain reaction strategy described recently (13) with minor changes. Positive amplification does not differentiate between homozygous and heterozygous carriers. Carrier status was thus recorded as binary variable (carrier vs noncarrier). Details on the assay establishment and conditions are given in the Supplemental Methods and in Supplemental Table 2. *LPA* KIV-2 4925G>A carrier status was available from previous projects (11,13).

### Study Description

The GCKD (German Chronic Kidney Disease) study is an ongoing prospective cohort study comprising 5,217 White patients with moderately severe chronic kidney disease. The design has been described previously (14). Study characteristics are given in the Supplemental Methods and in Supplemental Table 3. The study was approved by the ethics committees of all participating institutions and registered in the national registry for clinical studies (DRKS 00003971). Data for 4733G>A were available for 4,907 participants. Lp(a) isoforms and KIV-2 variant data on 4733G>A and 4925G>A were available from 4,673 participants. Genotypes for rs10455872, rs41272114, and rs3798220 were available for 4,578 of these. Informed consent was obtained from all participants. All studies were performed in accordance with the Declaration of Helsinki.

### Lp(A) Phenotyping

Lp(a) concentrations and apo(a) isoforms were determined by a well-established enzyme-linked immunosorbent assay and by Western blot, respectively (Supplemental Methods). All Western blots were inspected by the same researcher. In heterozygous individuals isoform 1 denotes the smaller (fewer KIV repeats than isoform 2) and isoform 2 the larger apo(a) isoform (more KIV repeats than isoform 1) observed on the Western blot. The relative contribution of isoform 1 to the total blot signal was quantified visually, providing a semiquantitative ranking of the relative expression levels. This defined the dominant isoform, which contributes 50% or more to the Western blot signal, and the nondominant isoform. In UK Biobank Lp(a) was quantified using a Randox AU5800 system (UK Biobank Data-Field 30790) (details in Supplemental Methods).

### Public Data Sets And The 1000 Genomes Project

We tested 66 single nucleotide polymorphisms (SNPs) by chi-square test for correlation with 4733G>A carrier status. The SNPs comprised all independent genome-wide significant proxy SNPs of the LPA gene region from our recent genome-wide association study (GWAS) (combined list from Supplemental Tables 6 and 8 from Mack et al [15]) that were represented in the Haplotype Reference Consortium-imputed data set (n = 64), plus rs3798220 and rs10455872. Linkage disequilibrium measures were assessed using the R package genetics, assuming that all 4733G>A carriers were heterozygous (details on selection of the proxy SNP in Supplemental Methods). The proxy SNP rs75692336 was used to tag KIV-2 4925G>A as assessed in Coassin et al (11).

The Type 2 Diabetes Knowledge Portal (16) was used to query the phenome effects of the proxy SNP for 4733G>A. The effect of carrying both 4733G>A and 4925G>A on CAD was assessed in UK Biobank with age-as-time-scale Cox proportional hazards regression using the proxy SNP combination rs75692336 (for 4925G>A) and rs6938647 (for 4733G>A) (UK Biobank application 62905). CAD was defined according to van der Harst et al (17) (ICD-10 codes I21-I25). Analyses were restricted to Whites. Details on survival analysis and assessment of the 1000 Genomes Project populations are available in the Supplemental Methods.

### Minigene Assay

We generated *LPA*-pSPL3 minigenes containing the wild type and the 4733G>A mutation as described previously (11). The minigene vector contains both KIV-2 exons, the intron between them plus 475 bp and 621 bp flanking sequence (Supplemental Figure 1). Technical details and in silico predictions are given in the Supplemental Methods.

### Statistical Methods

Because the distribution of Lp(a) is highly skewed, quantile regression, modeling the conditional median of Lp(a), was used to test the association of 4733G>A, 4925G>A, and the combined carrier status with Lp(a) values (using R package quantreg) (18). To derive the proportion of explained variance (r^2^), linear models were applied on inverse normally transformed Lp(a) levels. Relative importance of the covariates was assessed with the R package relaimpo (19) (pmvd metric). It calculates r^2^ of all individual covariates based on weighted averaging over sequential r^2^ values. The sum adds up to the total r^2^ of the model. One thousand bootstrapping runs were used to calculate the CIs. Pearson chi-square test was used to analyze frequency distributions. To compare Lp(a) values between 2 groups, the Wilcoxon test was used. The proxy SNP approach and survival analysis in UK Biobank are described in the section regarding public data sets.

## Results

### Assay Validation And Performance

The allele-specific polymerase chain reaction assay (13) showed no amplification at 0% mutant fraction, while still clearly detecting mutation fractions as low as 0.5% (Supplemental Figure 2). This easily allows the detection of the mutation if it is only available in 1 of the maximal possible sum of 80 to 90 KIV-2 repeats (1/90 = 1.1%). It correctly confirmed all 59 GCKD samples in which 4733G>A carrier status had been determined previously by ultra-deep next-generation sequencing (23 positives and 36 negatives) (12). Genotyping success rate was 99.96%.

### Lpa Kiv-2 4733G>A Is Associated With Reduced Lp(A) And Explains 12% Of Lp(A) Variance

We detected the KIV-2 4733G>A variant in 1,788 of 4,673 individuals (carrier frequency 38.26%). This corresponds to a minor allele frequency of 22.4% assuming Hardy-Weinberg equilibrium.

In a quantile regression analysis adjusted for age, sex, estimated glomerular filtration rate, and isoform 1, carrier status of 4733G>A was associated with 13.6 mg/dL (95% CI: 12.5-14.7 mg/dL) lower Lp(a) concentrations and explained 9.6% of Lp(a) variance (P < 0.0001) (Table 1). Addition of the carrier status of the high impact variant KIV-2 4925G>A (11) as a further covariate refined the effect to 12.6 mg/dL lower concentrations (95% CI: 11.4-13.9 mg/dL; P < 0.0001) and increased the Lp(a) variance explained by KIV-2 4733G>A to 11.9%. The complete regression model explained 46.1% of Lp(a) variance (Table 1). Further inclusion of the null allele rs41272114 (20) increased the explained variance of the model to 48.0% without blunting the effect and the variance explained by 4733G>A (Table 1). Rs41272114 occurs in a similar isoform range (≈27-33 KIV) (13) and thus could have been the causal variant underlying the association signal of 4733G>A. For comparison, another high impact KIV-2 variant (4925G>A) (11) shows a stronger effect (β= −22.6 mg/ dL) but explains only 4% to 7% of the Lp(a) variance in the population caused by the lower carrier frequency and restriction to isoforms 19 to 25 (Supplemental Table 4). Addition of *LPA* SNPs rs10455872 and rs3798220 (21) reduced the effect of 4733G>A to −8.8 mg/dL because these SNPs tag small isoforms with high Lp(a).

KIV-2 4733G>A provided the second strongest relative contribution to the total r^2^ of the model besides the apo(a) isoform 1 followed by 4925G>A, rs10455872, rs3798220, and rs41272114 (Supplemental Table 5).

### Variant 4733G>A Is Found Predominantly In Medium To Large Isoforms

**Figure 1:**
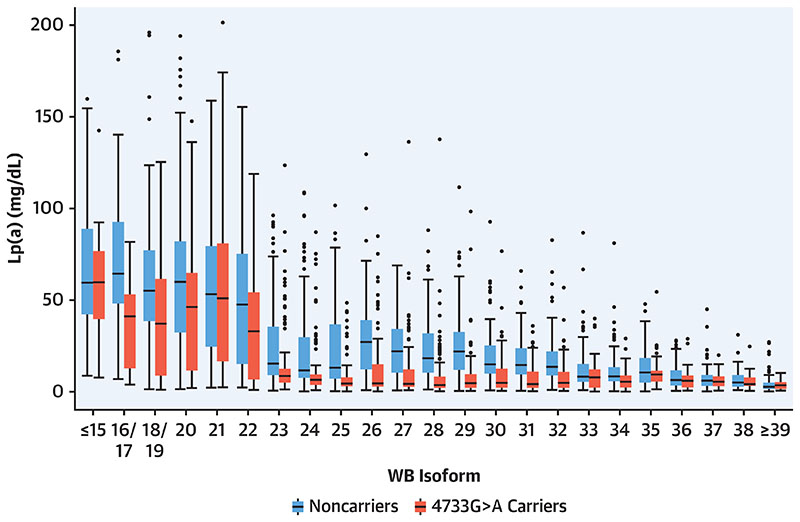
Lp(a) Concentrations by Carrier Status and Isoform 1 in GCKD The 4733G>A variant lowers lipoprotein(a) (Lp(a)) over the complete isoform range. Scale restricted to <210 mg/dL for better representation. Isoform grouping was done to have $20 in each group. Number of carriers per group are given in Supplemental Table 6. The same figure restricted to individuals that do not carry 4925G>A is shown in Supplemental Figure 3. GCKD = German Chronic Kidney Disease study; WB = Western blot.

**Figure 2:**
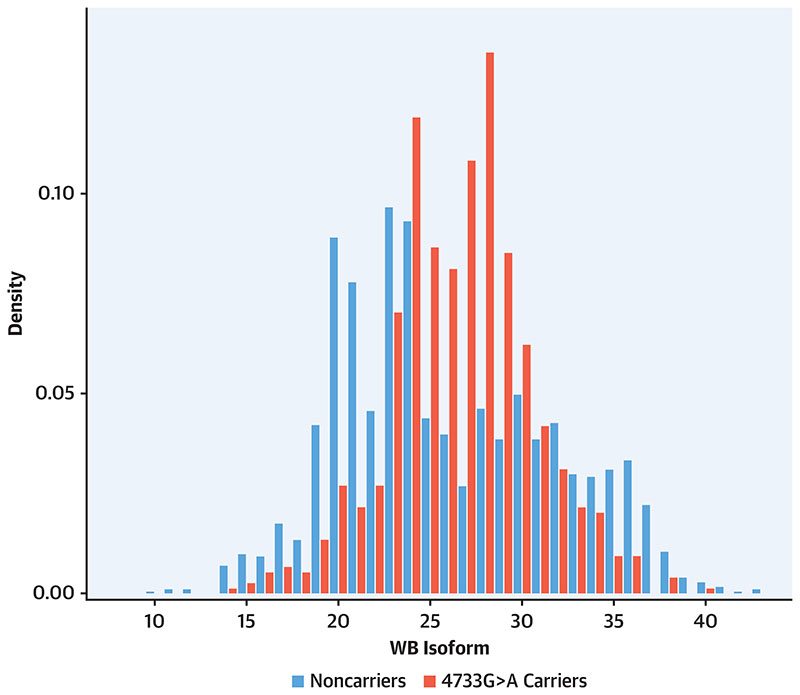
Distribution of Isoform 1 by 4733G>A Carrier Status in GCKD The 4733G>A variant is predominantly expressed in isoforms 24 to 33. Plot is restricted to individuals who express 2 isoforms in plasma. Abbreviations as in Figure 1.

KIV-2 4733G>A was observed in almost all isoform sizes and lowers Lp(a) in all isoform groups (Figures 1 and 2, Supplemental Table 6), but it shows some preferential association with alleles 24 to 33 KIV repeats (Figure 2). Exclusion of carriers of 4925G>A did not change the results (Supplemental Figure 3). Supplemental Figure 4 illustrates how the expression behavior of the apo(a) isoforms is influenced by the 4733G>A variant. As expected, in noncarriers expressing 2 apo(a) isoforms the smaller isoform (isoform 1) was commonly also the dominant isoform (ie, more abundant) (Supplemental Figure 4B). Conversely, in 4733G>A carriers the proportion of smaller isoforms that are also the dominant ones was significantly reduced (54.2% vs. 80.5%; P < 0.0001) (Supplemental Figures 4B and 4C), resulting in a switch of the isoform dominance in many individuals. This effect was most evident in isoforms 24 to 33. This suggests that 4733G>A lowers the expression of the allele on which it is located. Accordingly, isoform-specific Lp(a) concentrations are reduced in carriers (Supplemental Figure 5). Additionally, we noted a clear preponderance of individuals expressing only 1 isoform in plasma in the 4733G>A carriers vs noncarriers (58.7% vs 40.9%; P < 0.0001). This may indicate that 4733G>A lowers expression to values below the detection limit when 4733G>A occurs on an already lowly expressing allele.

### Modification Of Splicing In Vitro

**Figure 3:**
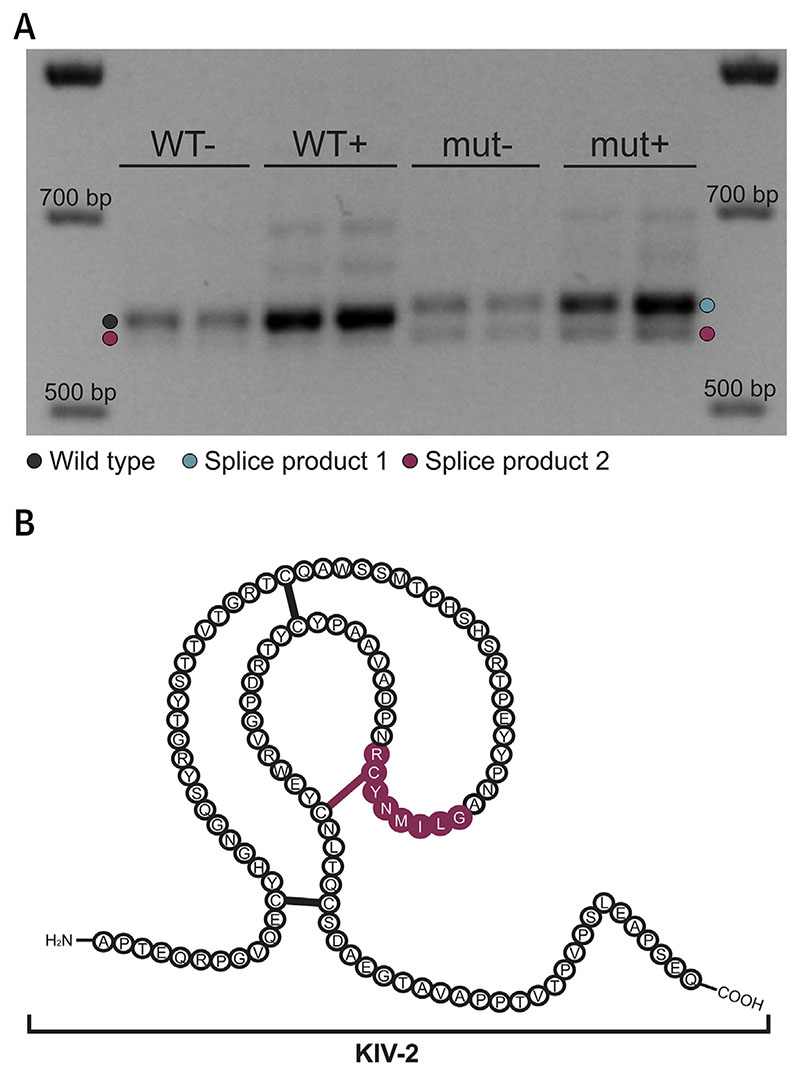
KIV-2 4733G>A Modifies Splicing, Causing Deletion of 1 Structure-Determining Cysteine **(A)** Representative gel of the minigene reverse-transcriptase polymerase chain reaction products (wild type [WT], mutant [mut], · puromycin) from 5 biological replicates with 2 technical replicates each (Supplemental Figure 6), showing different splicing behavior as described in the text. **(B)** Kringle IV type 2 (KIV-2) structure according to Guevara et al (37) with the amino acids and the disulfide bond (magenta) abolished by activation of splice site 3 (Supplemental Figure 8). bp = base pair(s).

The 4733G>A variant is located 11 bp upstream of the second KIV-2 exon in the intronic AG-exclusion zone (22). Bioinformatic predictions concordantly propose the generation of a novel splice acceptor 1 bp downstream of 4733G>A while abolishing the wild-type acceptor site (Supplemental Table 7). The LPA-pSPL3 minigene used in Coassin et al (11) (Supplemental Figure 1) recapitulated precisely the bioinformatic predictions, abolishing the wild-type splice site and creating 2 new splicing patterns (Figure 3, Supplemental Figures 6–8). It caused a 9-bp intron retention (splice site 2 in Supplemental Figure 8; splice product 1 in Figure 3) adding Ala-Ile-Ser-Ser between the KIV-2 exons (including the last exonic triplet) and increased the use of a cryptic splice acceptor located 24 bp in the exon following 4733G>A. This deletes 8 amino acids, including a KIV-2 structure-determining cysteine (Figure 3, splice site 3 in Supplemental Figure 8, splice product 2 in Figure 3). The latter product was present at a minor level in the wild-type constructs but was enhanced in the mutant ones (Supplemental Figure 6).

### Lpa Kiv-2 4733G>A and 4925G>A Jointly Induce Very Low Lp(A) Concentrations

**Figure 4:**
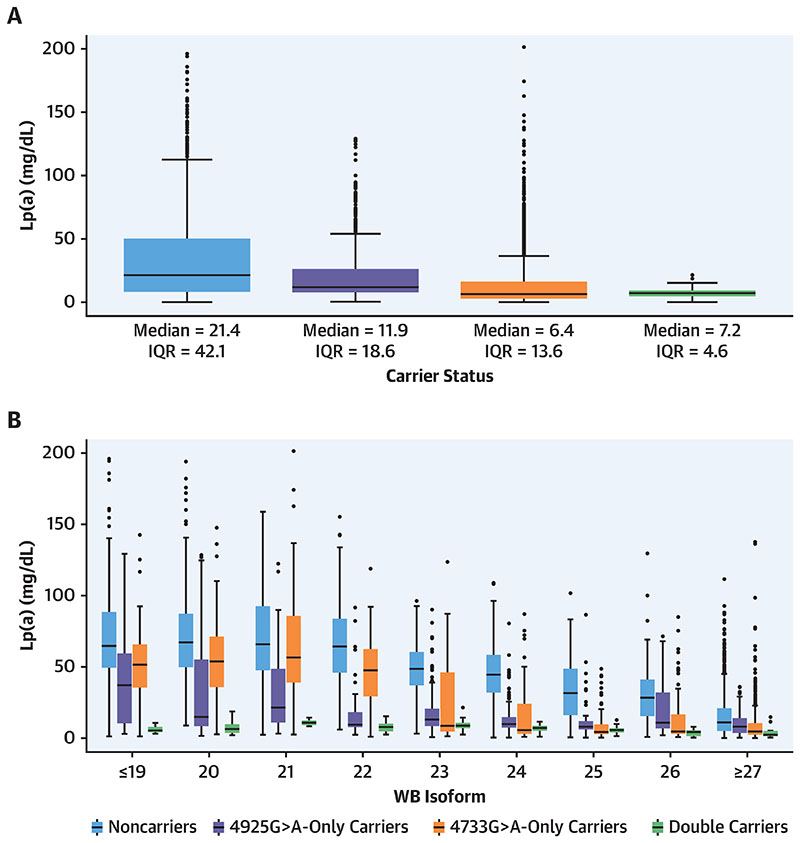
Lp(a) Concentrations in Carriers of 4733G>A and 4925G>A in GCKD **(A)** The graph shows Lp(a) concentrations in the 4 groups. **(B)** The graph shows Lp(a) concentrations that are additionally grouped by the isoform 1. Both variants lower Lp(a) but virtually no variability is left in the double carriers. Isoform grouping was done to have $5 per group. Scale restricted to <210 mg/dL for better representation. Numbers per isoform stratum are given in Supplemental Table 8. IQR = interquartile range; other abbreviations as in Figure 1.

A total of 216 GCKD participants (4.6%) carried both KIV-2 4733G>A and KIV-2 4925G>A (11) (Supplemental Table 8). Both SNPs independently lower Lp(a) (Figure 4A, Table 2), but still present a considerable range of Lp(a) values, mostly caused by the contribution of the second nonmutated allele (Figure 4A). In contrast, individuals carrying both variants show a similar median Lp(a) as individuals carrying only 4733G>A or 4925G>A, but with almost no Lp(a) variability left (Figure 4A), indicating that the SNPs are likely located in trans and blunt both alleles (but without causing null alleles). Such a compound heterozygosity reduces Lp(a) over the complete range of isoforms (Figure 4B, Central Illustration). In a regression analysis adjusted for age, sex, and isoform 1, double carrier status was associated with a Lp(a) reduction of 31.8 mg/dL (95% CI: 30.2-33.3 mg/dL; P < 0.0001). This was still a reduction of 17.5 mg/dL (95% CI: 16.0-18.8 mg/dL; P < 0.0001) compared with that of noncarriers for both variants after additional adjustment for rs10455872, rs3798220, and rs41272114 (Table 2).

### Association With Reduced Risk in Uk Biobank

**Figure 5:**
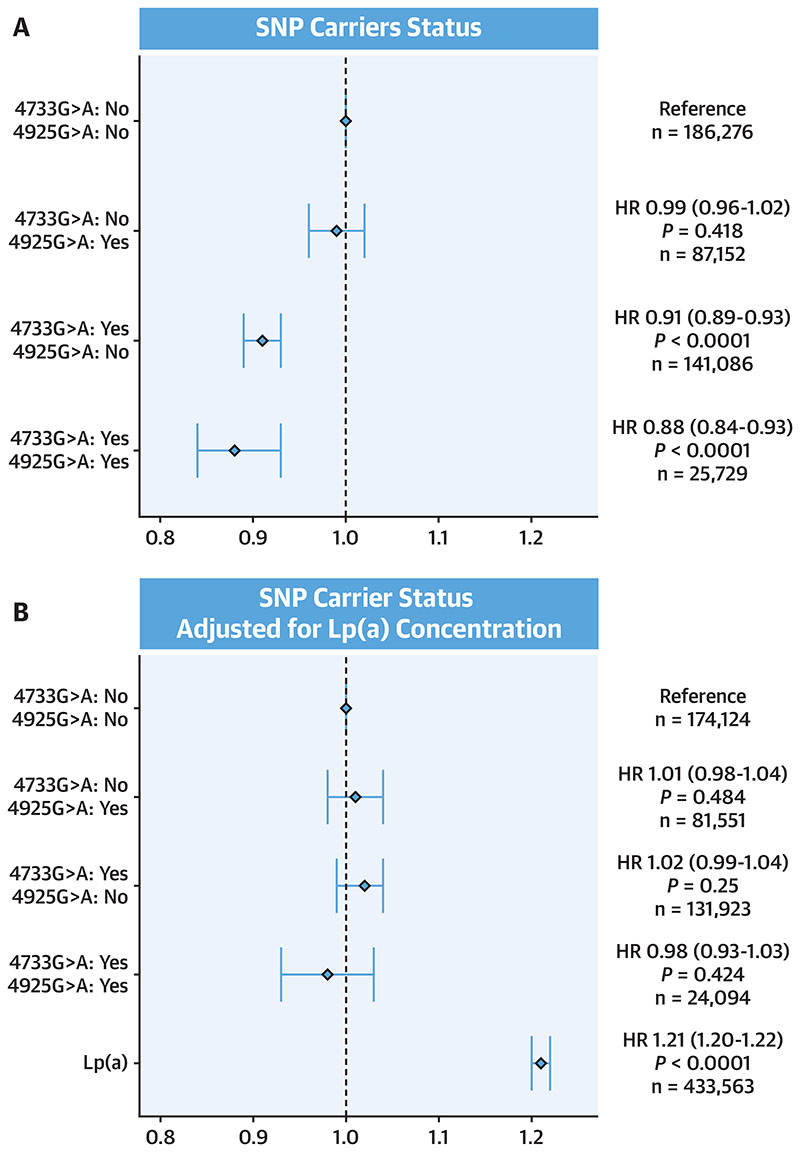
HR for CAD Riskin UK Biobank **(A)** Model is adjusted for sex. **(B)** Model is adjusted for sex and inverse-normal transformed lipoprotein(a) (Lp(a)) concentration. Plot is restricted to individuals with Lp(a) measurements available. Status refers to the presence (yes) or absence (no) of the proxy single nucleotide polymorphisms (SNPs) rs6938647/rs75692336 for 4733G>A/4925G>A. The noncarriers of proxy SNPs of both variants make up the reference group. Age is taken as time scale (model with time-on-study shown in Supplemental Figure 10). HR for Lp(a) concentration is given for a 1-unit increase of the inverse-normal transformed Lp(a) concentration. CAD = coronary artery disease.

The location of 4733G>A (and 4925G>A) in the KIV-2 prevents the lookup of their impact in public data sets. We therefore screened the hits of our recent GWAS (15) on Lp(a) for linkage disequilibrium with 4733G>A (Supplemental Table 9). This approach has previously successfully identified rs75692336 as proxy for 4925G>A(11).The search for the best proxy SNP for 4733G>A is described in the Supplemental Table 10 and revealed rs6938647 (D^0^ = 0.931, r^2^ = 0.682). This proxy SNP (minor allele frequency of 21.0% in CARDIoGRAMplusC4D-UK Biobank) showed highly significant association with CAD in a recent meta-analysis of the CARDIoGRAM and UK Biobank data (17)(n= 547,261) and resulted in a 7.4% (95% CI: 6.0%-8.7%) lower CAD risk (P < 0.0001). The effect of carrying 4733G>A, 4925G>A and compound heterozygosity on prospective CAD risk was assessed by Cox regression in UK Biobank (n = 440,234) using the proxy combination rs75692336 (for 4925G>A) and rs6938647 (for 4733G>A). Effect size on Lp(a) concentration in UK Biobank and GCKD study was similar (Supplemental Figure 9, Figure 4A). As shown previously (11), 4925G>A alone did not reduce CAD risk in the whole population, because its effect is restricted to LMW carriers. In contrast, compared with non-carriers of both proxy SNPs, carriers of 4733G>Ahada 9% lower risk of CAD in the survival analysis (HR: 0.91; 95% CI: 0.89-0.93; P < 0.001) and compound heterozygotes of 4733G>A and 4925G>A had a 12% lower risk of CAD (HR: 0.88; 95% CI: 0.84-0.93; P < 0.001) (Figure 5A, Central Illustration). As expected from previous publications (23,24), the proxy SNPs were not significantly associated with CAD risk anymore, when the analysis was adjusted for the Lp(a) concentration (Figure 5B).

### Allele Frequency Shows Ethnic Differences

We assessed its frequency in our recent catalog of genetic variation in the KIV-2 (12) in the 1000 Genomes Project. The 4733G>A variant was found in the European, Latin American (Admixed American), and South Asian continental groups, whereas it was not detected in East Asians and was very rare in Africans (Supplemental Tables 11–13). The carrier frequency varies considerably from 1.5% in Africans to 35.1% Europeans. Among the African populations, it was indeed observed only in populations with some potential of admixture (African Caribbeans in Barbados and Americans of African Ancestry in the Southwestern United States).

## Discussion

Whereas important progress has been made recently in understanding the Lp(a)-mediated cardiovascular risks (1,2), the details of its genetic regulation and especially the mechanisms underlying its variance are still largely unclear. The 200-fold range observed within the same isoform size groups indicates that genetic variants may modulate the impact of the apo(a) isoforms but very few are known. Because the KIV-2 can encompass a large portion of codingsequence (up to 70%), it may contain several functional variants missed so far. The recently described variant LPA KIV-2 4925G>A is such a variant and explains some peculiarities of the Lp(a) trait, but it is restricted to a rather narrow isoform range (11).

In this work we have identified a second very frequent KIV-2 variant (named 4733G>A).It is present in nearly 40% of the population but has not been investigated in detail earlier because of its previously inaccessible location in the KIV-2 repeat. It is associated with 13.6 mg/dL lower Lp(a) and, because of its very high frequency, it explains z10% of Lp(a) variance, making it the strongest determinant of Lp(a) concentrations besides the apo(a) isoform size.

The 4733G>A variant is found in nearly every isoform size, albeit with some preponderance in isoforms 24 to 33. Of note, we observed a switch in the dominant isoform patterns in the Western blots of 4733G>A carriers (explained in Supplemental Figure 4). This provides a molecular basis for the long-standing reports about exceptions in the dominance of the shorter isoforms in Western blots and suggests that the expression of the allele carrying 4733G>A is reduced. Accordingly, in silico predictions, location of the variant in the AG-exclusion zone region, and in vitro experiments concordantly propose that 4733G>A modulates splicing. By creating a new AG dinucleotide in the AG-exclusion zone, 4733G>A creates a new splice acceptor. Interestingly, both aberrations caused by 4733G>A create in-frame proteins, but the deletion abolishes 1 disulfide bond that creates the kringle structure. The Lp(a) reduction may thus be caused by altered secretion, folding, and/or processing of the protein. Similar effects have been recently shown for missense variants in LPA (25).

A very heterogeneous picture for the causes of the large variability of Lp(a) concentrations in the population emerges where several genetic variants— including modifiers of LPA expression, loss-of-function alleles (both reviewed in Schmidt et al (20)), splicing modification (11) and even missense variants impairing secretion (25)—independently modify Lp(a) concentrations. Based on variation patterns from >100,000 genomes, the GnomAD project (26) assigns a “probability of being intolerant for [loss-of-function] variants” (pLi) score (27) of 0 for LPA.This pLi score uses the ratio of observed vs ex-pected protein-truncating mutations in z126,000 exomes to infer the tolerance of a given gene for the occurrence of loss-of-function mutation. The low pLi score might suggest that atleast the lower part of the Lp(a) concentration range observed for each isoform might be caused by multiple lesions acting together.

Accordingly, 4733G>A/4925G>A compound heterozygotes, compared with the wild types, have on average 31.8 mg/dL lower Lp(a) concentrations and a 9-fold narrower interquartile range (42.1 vs 4.6 mg/dL). It is tempting to speculate that different frequencies of such variants might contribute to the interethnic differences in Lp(a). Indeed, similar to many other Lp(a)-lowering variants (4925G>A [11], R21X [13], rs41272114 [13], rs3798220 [20]) 4733G>A presents pronounced interethnic frequency differences. More genome data about non-White individuals are required to deepen these findings.

## Clinical Implications

Besides providing novel insights in the architecture of the Lp(a) trait, our findings have relevant clinical implications as shown in the Central Illustration. We show that the lifelong 13.6 mg/dL Lp(a) reduction associated with 4733G>A is associated with a significant decrease in CAD risk of 9% in a Cox regression analysis in UK Biobank. This suggests that lifelong exposure to even moderately reduced Lp(a) is associated with a detectable decrease in cardiovascular disease risk. This might even be an underestimation because this could be assessed only via an incomplete proxy SNP because variants in the KIV-2 region are not well captured by common genome-wide data. However, almost 90% of all proxy SNP carriers were also carriers of 4733G>A, providing a good estimate of the effects of 4733G>A itself.Importantly, the effect estimate for the compound heterozygosity of 4733G>A and 4925G>A might be attenuated because only about 76% of proxy haplotype carriers also carried both variants.

Another open question in clinical interpretation of Lp(a) is whether the isoform size provides any additional risk beyond the Lp(a) concentrations. After adjustment for the Lp(a) concentration, the carrier status for 4733G>A and 4925G>A was not significantly associated with CAD risk anymore. This indicates that their effect on CAD risk is mediated by the Lp(a) concentration, which is in line with previous publications (23,24). A differential effect of apo(a) isoforms has been discussed for cardiovascular disease risk in renal patients (28) and load of oxidized phospholipids (4). Also in diabetes research the impact of the isoform size per se rather than the Lp(a) concentrations is an ongoing discussion (29,30). The 4925G>A variant, which lowers Lp(a) selectively in LMW isoforms, has been used recently elegantly as an instrument to disentangle the Lp(a) concentrations from isoform size in a large Icelandic cohort (24). Given its high frequency, 4733G>A will be an even stronger instrument to further tackle this unresolved clinically very relevant question. Finally, we are now on the dawn of therapeutic inactivation of atherogenic genes by somatic gene editing, which could potentially provide a true single shot intervention for high-risk individuals (31–35). In the past decade, human genetics has been used to validate drug targets (36). In an analogous way, very frequent Lp(a)-lowering variants may represent safe targets to be mimicked by therapeutic gene editing.

## Study Strengths And Limitations

We have designed a robust assay for a variant in the largely unaddressed KIV-2 region, which makes up most of the *LPA* coding sequence, and we used the data not only to assess the impact on Lp(a) but also to provide proxy SNPs that are contained in GWAS data. Using large public data sets of CARDIoGRAMplusC4D and UK Biobank, we show a clear impact on CAD risk, underscoring the importance of even minor but lifelong Lp(a) reduction, and using the 1000 Genomes Project, we show that the genotypes of 4733G>A can be directly extracted from available sequence data, allowing replication and extension of our findings by others. Our study is limited by the complex linkage disequilibrium patterns in *LPA*. We assessed the linkage disequilibrium with all hits of our recent GWAS on Lp(a), but clearly we cannot completely rule out that a linkage disequilibrium with other unknown functional variants may account for some of the observed effects. However, we see the effect of 4733G>A in all isoform groups, it is not markedly modified by other SNPs and the splicing assays show a clear impact that matches bioinformatics predictions. We recognize that the allele-specific polymerase chain reaction strategy cannot differentiate homozygous and heterozygous carriers limiting exact estimation of effect size. We acknowledge that we inferred the allelic location of 4733G>A rather than determining it directly, because selecting a few carriers for pulsed-field electrophoresis-based assessment, as was done recently (11,13), would not be meaningful, given the high frequency of the variant. Finally, we are aware that our association results are limited to individuals of European ancestry and replication studies in populations of different ancestry are warranted. Our results from the 1000 Genomes Project may hopefully guide such studies.

## Conclusions

We describe a novel putative splicing modulator in *LPA* that is the second strongest genetic effect on Lp(a) variance, at least in a White population. It provides insights in the genetic architecture behind the large variance of Lp(a) and highlights how even moderate lifelong reductions in Lp(a) concentration results in a clinical benefit.

## Supplementary Material

For supplemental methods, references, figures, and tables, please see the online version of this paper.

Supplementary File

## Figures and Tables

**Table 1:** Quantile Regression Analysis Between 4733G>A Carrier Status and Lp(a) Levels

Model and Adjustment	β (95% CI)	P Value	r^2^ by Model/4733G>A^a^
1. Age, sex, eGFR, and isoform1	−13.6(−14.7 to −12.5)	<0.0001	0.392/0.096
2. As model 1 plus 4925G>A	−12.6 (−13.9 to −11.4)	<0.0001	0.461/0.119
3. As model 2 plus rs41272114	−12.7 (−13.9 to −11.4)	<0.0001	0.480/0.126
4. As model 3 plus rs10455872 and rs3798220	−8.8 (−9.7 to −8.0)	<0.0001	0.511/0.099

a Variance explained (r^2^) derived from linear model on inverse normal transformed Lp(a) concentrations.

CI = confidence interval; eGFR = estimated glomerular filtration rate; Lp(a) = lipoprotein(a).

**Table 2:** Quantile Regression Analysis of the Double Carrier Status (4733G>A and 4925G>A) on Lp(a) Concentrations

Carrier Status 4733G>A/4925G>A	n	β (95% CI)	*P* Value	r^2^ by Model/Carrier Status^a^
Model 1. Adjusted for age, sex, eGFR, and isoform 1				0.462/0.165
Yes/yes	216	−31.8 (−33.3 to −30.2)	<0.0001	
Yes/no	1,572	−16.5 (−17.6 to −15.3)	<0.0001	
No/yes	724	−26.6 (−28.3 to −25.0)	<0.0001	
Model 2. Adjusted as in model 1 plus rs41272114				0.480/0.172
Yes/yes	216	−32.2 (−33.7 to −30.7)	<0.0001	
Yes/no	1,572	−16.4 (−17.6 to −15.2)	<0.0001	
No/yes	724	−26.8 (−28.3 to −25.3)	<0.0001	
Model 3. Adjusted as in model 2 plus rs3798220 and rs41272114				0.512/0.117
Yes/yes	216	−17.5 (−18.8 to −16.0)	<0.0001	
Yes/no	1,572	−10.9 (−11.9 to −9.8)	<0.0001	
No/yes	724	−13.2 (−14.6 to −11.9)	<0.0001	

Carrier status refers to the presence (yes) or absence (no) of the 2 variants (given as 4733G>A/4925G> A). Noncarriers of both variants make up the reference group.

a Variance explained (r^2^) derived from linear model on inverse normal transformed Lp(a) concentrations. Abbreviations as in Table 1.

## Perspectives

### Competency In Medical Knowledge

Frequently occurring variants in the LPA gene dissociate apo(a) isoforms from blood Lp(a) concentration, limit the predictive power of the apo(a) isoforms, and make Lp(a) concentrations a more accurate measure of coronary disease risk.

### Translational Outlook

These variants that modify Lp(a) concentrations may represent novel therapeutic targets to reduce atherosclerotic risk.

## Abbreviations And Acronyms

apo(a): apolipoprotein(a)
bp: base pair(s)
CAD: coronary artery disease
GWAS: genome-wide association study
KIV-2: kringle IV type 2
LMW: low molecular weight
Lp(a): lipoprotein(a)
pLi: probability of being intolerant for loss-of-function variants (score)
SNP: single nucleotide polymorphism
