# Supplementary material for: Frequent LPA KIV-2 Variants Lower Lipoprotein(a) Concentrations and Protect Against Coronary Artery Disease
Source: J Am Coll Cardiol. Author manuscript; Available in PMC 2022 Sep 15. (PMC7613585; doi:10.1016/j.jacc.2021.05.037)
Supplement: Supplementary File [file EMS152741-supplement-Supplementary_File.pdf]

Supplemental materials to

Frequent *LPA* KIV-2 variants lower lipoprotein(a) concentrations and protect against coronary artery disease

Johanna F. Schachtl-Riess, MSc[a], Azin Kheirkhah, MSc[a], Rebecca Grüneis, MSc[a], Silvia Di Maio, MSc[a], Sebastian Schoenherr, PhD[a], Gertraud Streiter, BSc[a], Jamie Lee Losso, BSc[a], Bernhard Paulweber, MD[b], Kai-Uwe Eckardt, MD[c,d], Anna Köttgen, MD MPH[e], Claudia Lamina, PhD[a], Florian Kronenberg, MD[a*], Stefan Coassin, PhD[a*] for the GCKD investigators

* contributed equally

[a] Institute of Genetic Epidemiology, Department of Genetics and Pharmacology, Medical University of Innsbruck, Innsbruck, Austria
[b] First Department of Internal Medicine, Paracelsus Private Medical University, Salzburg, Austria
[c] Department of Nephrology and Hypertension, Friedrich-Alexander Universität Erlangen-Nürnberg, Erlangen, Germany and German Chronic Kidney Disease study
[d] Department of Nephrology and Medical Intensive Care, Charité—Universitätsmedizin Berlin, Berlin, Germany
[e] Institute of Genetic Epidemiology, Faculty of Medicine and Medical Center, University of Freiburg, Freiburg, Germany

Current GCKD Investigators and Collaborators with the GCKD Study are:

University of Erlangen-Nürnberg: Kai-Uwe Eckardt, Heike Meiselbach, Markus P. Schneider, Mario Schiffer, Hans-Ulrich Prokosch, Barbara Bärthlein, Andreas Beck, André Reis, Arif B. Ekici, Susanne Becker, Dinah Becker-Grosspitsch, Ulrike Alberth-Schmidt, Birgit Hausknecht, Anke Weigel;

University of Freiburg: Gerd Walz, Anna Köttgen, Ulla T. Schultheiß, Fruzsina Kotsis, Simone Meder, Erna Mitsch, Ursula Reinhard;

RWTH Aachen University: Jürgen Floege, Turgay Saritas;

Charité, University Medicine Berlin: Elke Schaeffner, Seema Baid-Agrawal, Kerstin Theisen;

Hannover Medical School: Hermann Haller, Jan Menne;

University of Heidelberg: Martin Zeier, Claudia Sommerer, Johanna Theilinger;

University of Jena: Gunter Wolf, Martin Busch, Rainer Paul;

Ludwig-Maximilians University of München: Thomas Sitter;

University of Würzburg: Christoph Wanner, Vera Krane, Antje Börner-Klein, Britta Bauer;

Medical University of Innsbruck, Institute of Genetic Epidemiology: Florian Kronenberg, Julia Raschenberger, Barbara Kollerits, Lukas Forer, Sebastian Schönherr, Hansi Weissensteiner;

University of Regensburg, Institute of Functional Genomics: Peter Oefner, Wolfram Gronwald;

University of Bonn, Institute of Medical Biometry, Informatics and Epidemiology, Medical Faculty: Matthias Schmid, Jennifer Nadal.

Content

Supplemental Figures 3
Supplemental Tables 15
Supplemental Methods 30
Variant naming 30
Assay development 30
Assay validation 30
Description of the GCKD study 30
ELISA and Western blot in GCKD 31
Look up in public datasets 31
Frequency in 1000 Genomes 31
Survival analysis and Lp(a) concentrations in UK Biobank 31
Bioinformatic predictions 32
Minigene assay 32
References 33

Supplemental Figures

Supplemental Figure 1: Minigene structure.

The 4733G>A variant is located 11 bp upstream of the 5' exon boundary. The length of the expected transcript if all four exons are spliced correctly is known (602 bp). Splicing aberrations are detected by PCR products that differ in length and/or sequence from the transcript that is expected.

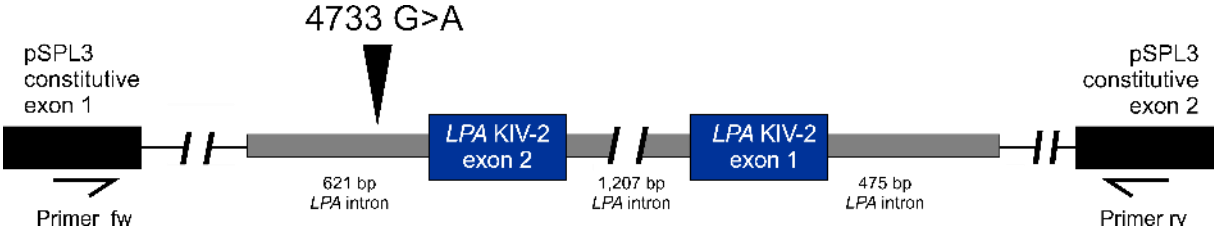

Supplemental Figure 2: Dilution series of mutant in wild type plasmid for asPCR 4733G>A.

Varying percentages of pSPL3 containing the 4733G>A mutation was diluted with wild type pSPL3. 69 fg of DNA was used per reaction (corresponding to about the amount of genome copies present in 20 ng genomic DNA). The percentage of mutant plasmid is given above each sample pair. The lane indicated as “0%” contains only the wild type plasmid. The primer does not produce an amplicon at 0% mutation level while it shows an amplicon at 0.5% mutation level.

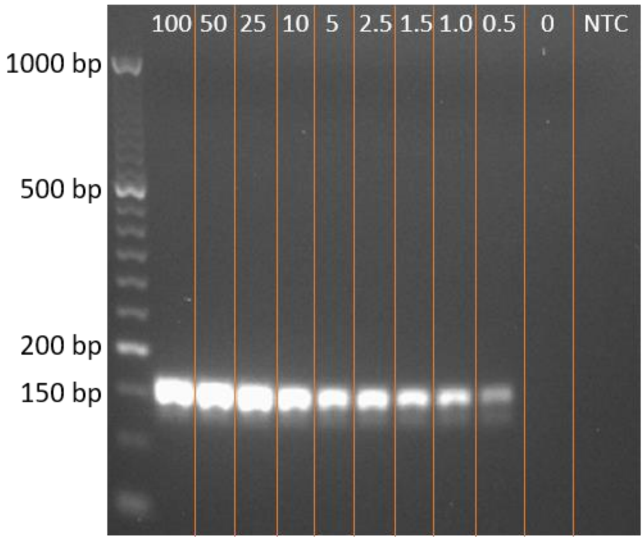

Supplemental Figure 3: Lp(a) concentrations of 4733G>A carriers and non-carriers over the whole isoform range, restricted to individuals who do not carry the 4925G>A variant.

This plot is restricted to individuals who do not carry the 4925G>A variant. Lp(a) concentration in mg/dL in non-carriers of 4733G>A (light blue) and carriers (orange) grouped by the number of KIV repeats on the smaller expressed isoform. Levels above 210 mg/dL are not shown for better representation. Absolute number of carriers per isoform stratum are given below each boxplot. Isoform grouping was done to have n≥20 in each group prior to subsetting for non-carriers of 4925G>A.

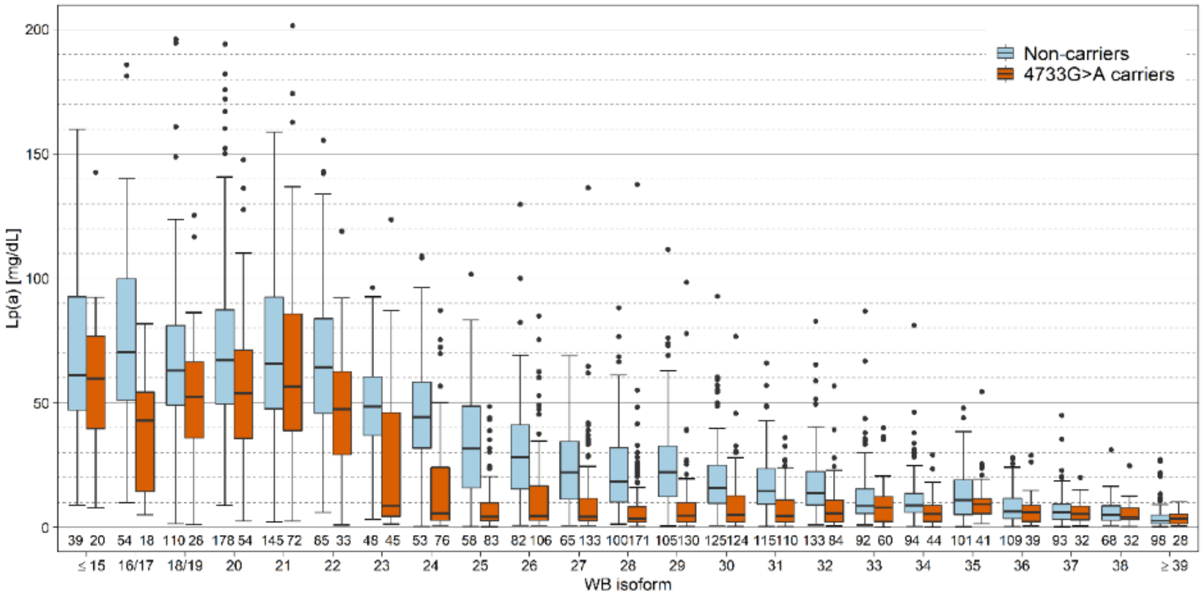

Supplemental Figure 4 (see next page): Dot plots of isoform dominance patterns in carriers and non-carriers of 4733G>A. The variant 4733G>A induces a switch in the isoform dominance. In non-carrier the shorter isoform is mostly also the dominant one, as would be expected. Conversely, in carriers of 4733G>A it can be seen that, especially in the range of isoform size 24 - 33, the shorter isoform is no more the dominant one, because 4733G>A likely reduces its expression levels. Which isoform is the dominant one was deduced from Western blots (WB).

(A) Guide for interpreting panel B and C;
(B) Isoform size and isoform dominance patterns in non-carriers of 4733G>A (n = 1216);
(C) Isoform size and isoform dominance patterns in carriers of 4733G>A (n = 613).

For each individual, the dominant isoform is colored in red and the non-dominant one in blue. Plots only include individuals who express two isoforms in plasma and do not carry 4925G>A.

Panel A) The dot plots show size of the isoforms in each individual. The figures are restricted to individuals that presented two isoforms in Western Blot (isoform 1 and isoform 2). Only non-carriers of 4925G>A are shown because 4925G>A has been shown before to reduce allelic expression (1). The individuals are sorted along the x-axis, the y-axis shows the isoform size (two dots on y-axis per individual). The individuals are ordered according to the size of Isoform 1 and within each isoform 1 size group, then by the size of isoform 2. The dominant isoform is colored in red, the non-dominant isoform in blue (in the example isoform 37 and 39, respectively).

Panel B) shows the non-carriers of 4733G>A. In this plot, Isoform 1 is the dominant isoform for most individuals.

In contrast, in **Panel C)** (carriers of 4733G>A) the dominant isoform is mostly the isoform 2. This indicates that in non-carriers the dominant isoform is usually the smaller isoform (as expected (2)). Conversely, in carriers of 4733G>A the smaller isoform is often the one that is expressed to a lower instead of a higher extend (i.e. it is the non-dominant isoform). Interestingly this applies mostly when isoform 1 is in the size range 24-33, while in samples with the smaller isoform <24 and especially <23 this switch in the dominance patterns of the isoforms is not seen and the non-dominant isoforms are still those >24, which here correspond to isoform 2. We assume that this is because the 4733G>A is predominantly (albeit not exclusively) associated with isoforms 24-33.

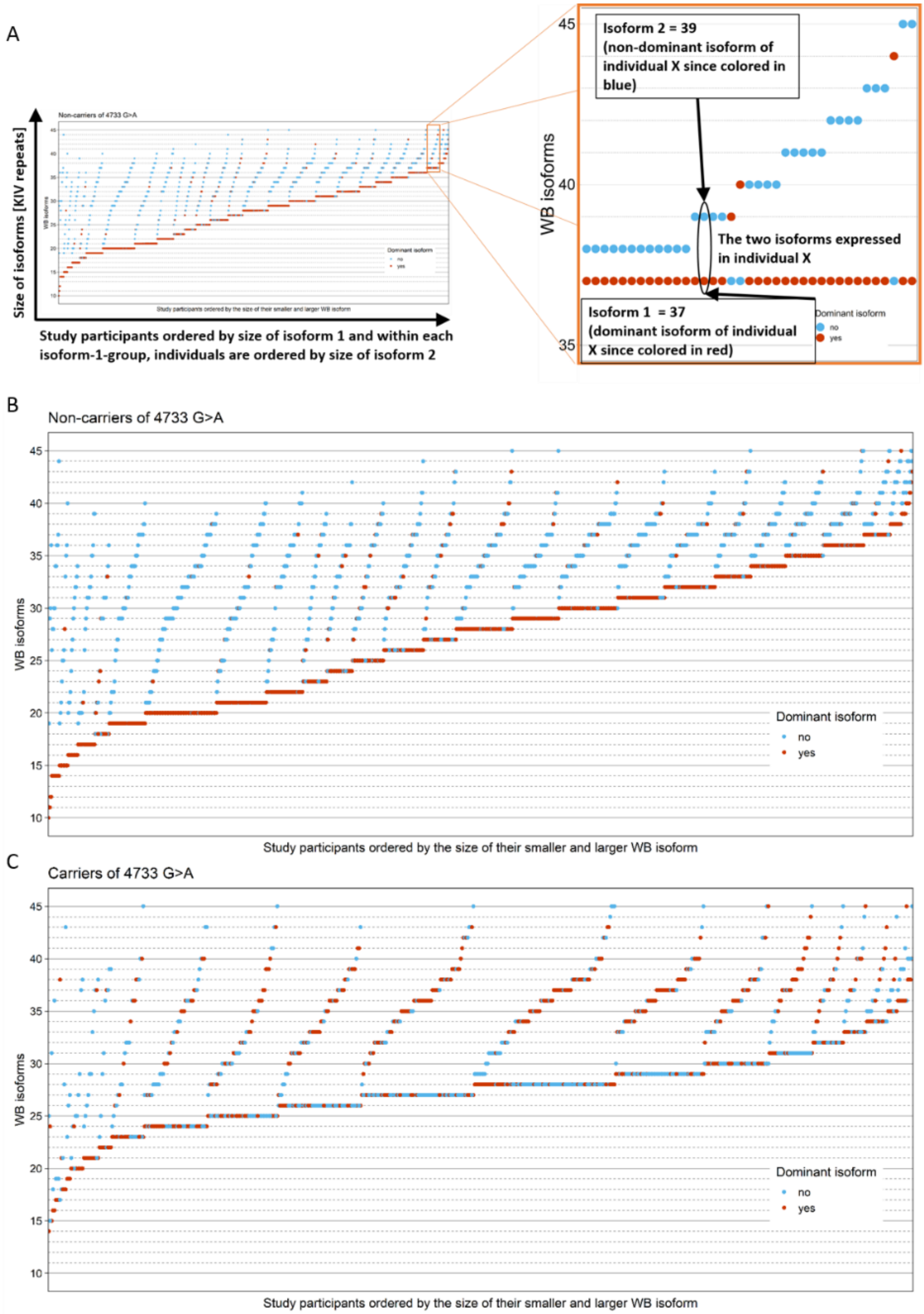
A
Size of isoforms [KIV repeats]
Study participants ordered by size of isoform 1 and within each isoform-1-group, individuals are ordered by size of isoform 2
WB isoforms
Isoform 2 = 39 (non-dominant isoform of individual X since colored in blue)
The two isoforms expressed in individual X
Isoform 1 = 37 (dominant isoform of individual X since colored in red)
Dominant isoform
no
yes
B
Non-carriers of 4733 G>A
WB isoforms
Dominant isoform
no
yes
Study participants ordered by the size of their smaller and larger WB isoform
C
Carriers of 4733 G>A
WB isoforms
Dominant isoform
no
yes
Study participants ordered by the size of their smaller and larger WB isoform

Supplemental Figure 5 (see next page): Isoform-specific Lp(a) concentrations of alleles of (A) 4733G>A and (B) 4925G>A carriers are reduced compared to alleles from non-carriers. However, these plots contain both alleles of variant carriers, whereas the 4733G>A and 4925G>A variants are mostly present only on one of the two alleles. Including only the allele of carriers that most likely carries the respective variant based on the isoform dominance pattern, shows that both variants lower the isoform-specific Lp(a) concentration, irrespective of the isoform size.

Blue: alleles from non-carriers of 4733G>A and 4925G>A. All plots are restricted to heterozygous carriers of isoforms since true homozygous carriers cannot be discerned from heterozygous carriers with one null allele. Lines represent smoothed conditional means based on the loess method (local polynomial regression fitting) in the *ggplot2* package in R and the gray shades represent 95%CIs. Values above 100 mg/dL are not shown for better visualization. Plots for 4733G>A (A, C) are restricted to non-carriers of 4925G>A to avoid confounding. Vice versa, plots for 4925G>A (B, D) are restricted to non-carriers of 4733G>A.

(A) Orange represents both alleles of 4733G>A carriers.
(B) Purple represents both alleles of 4925G>A carriers.
(C) Orange represents the 4733G>A carrying allele inferred by the dominance pattern. The other allele of 4733G>A carriers is excluded from this plot since homo- and heterozygous individuals cannot be discerned.
(D) Purple represents the 4925G>A carrying allele inferred by the dominance pattern. The other allele of 4925G>A carriers is excluded from this plot since homo- and heterozygous individuals cannot be discerned.

The isoform-specific Lp(a) concentrations were calculated by multiplying the total Lp(a) concentration of an individual with the relative expression of isoform 1 and isoform 2 derived from the Western blot data. The dominance pattern was used to infer the allelic location of the two functional variants, since it is expected that the non-dominant isoform carries the respective mutated variant. If isoform 1 is the dominant isoform in a variant-carrier, the variant is assumed to be on isoform 2. If isoform 1 (smaller expressed isoform) is the dominant isoform in a variant-carrier, the variant is assumed to be on isoform 2 (larger expressed isoform). If isoform 2 is the dominant isoform of a variant-carrier, the variant is assumed to be on isoform 1.

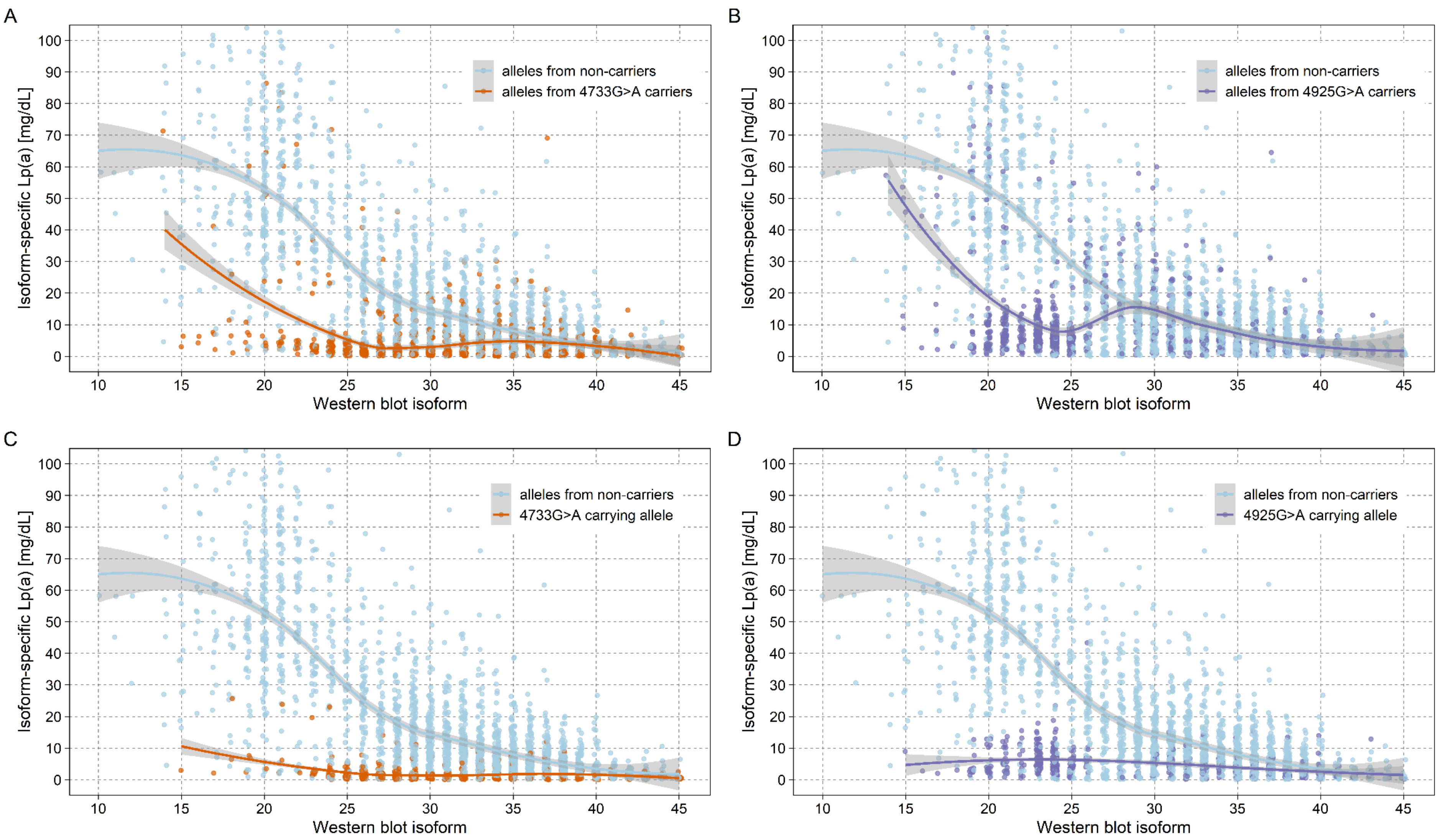
A
alleles from non-carriers
alleles from 4733G>A carriers
Isoform-specific Lp(a) [mg/dL]
Western blot isoform
B
alleles from non-carriers
alleles from 4925G>A carriers
Isoform-specific Lp(a) [mg/dL]
Western blot isoform
C
alleles from non-carriers
4733G>A carrying allele
Isoform-specific Lp(a) [mg/dL]
Western blot isoform
D
alleles from non-carriers
4925G>A carrying allele
Isoform-specific Lp(a) [mg/dL]
Western blot isoform

Supplemental Figure 6 (see next page): Minigene assay indicates splicing modulation.

A) Explanation of the observed effects as deduced by Sanger sequencing. Sanger sequencing trace with explanation is shown in Supplemental Figure 7. 4733G>A induced two different splice effects: splice product 1 uses a novel splice acceptor located one base downstream of 4733G>A leading to a 9 bp intron retention. Splice product 2 enhances a cryptic splice acceptor 34 bases downstream of 4733G>A, deleting 24 bases of KIV-2 exon 2. Usage of these latter splice acceptor is observed also in the wild type products but at much lower level. This is in line with the bioinformatic predictions shown in Supplemental Figure 8. Both changes are in-frame.

B-E) Reverse Transcriptase PCR images showing the effect of 4733G>A in five biological replicates with two technical replicates each. 4733G>A activates two cryptic splice sites. The upper band (blue dot) is the splice product 1, which includes additional intronic bases. The lower band (magenta dot) is the splice product 2, which deletes 24 exonic bases. The grey dot indicates the wild type product showing with a length between the two aberrant splice products. WT: wild type, mut: mutant, +/- P: puromycin addition.

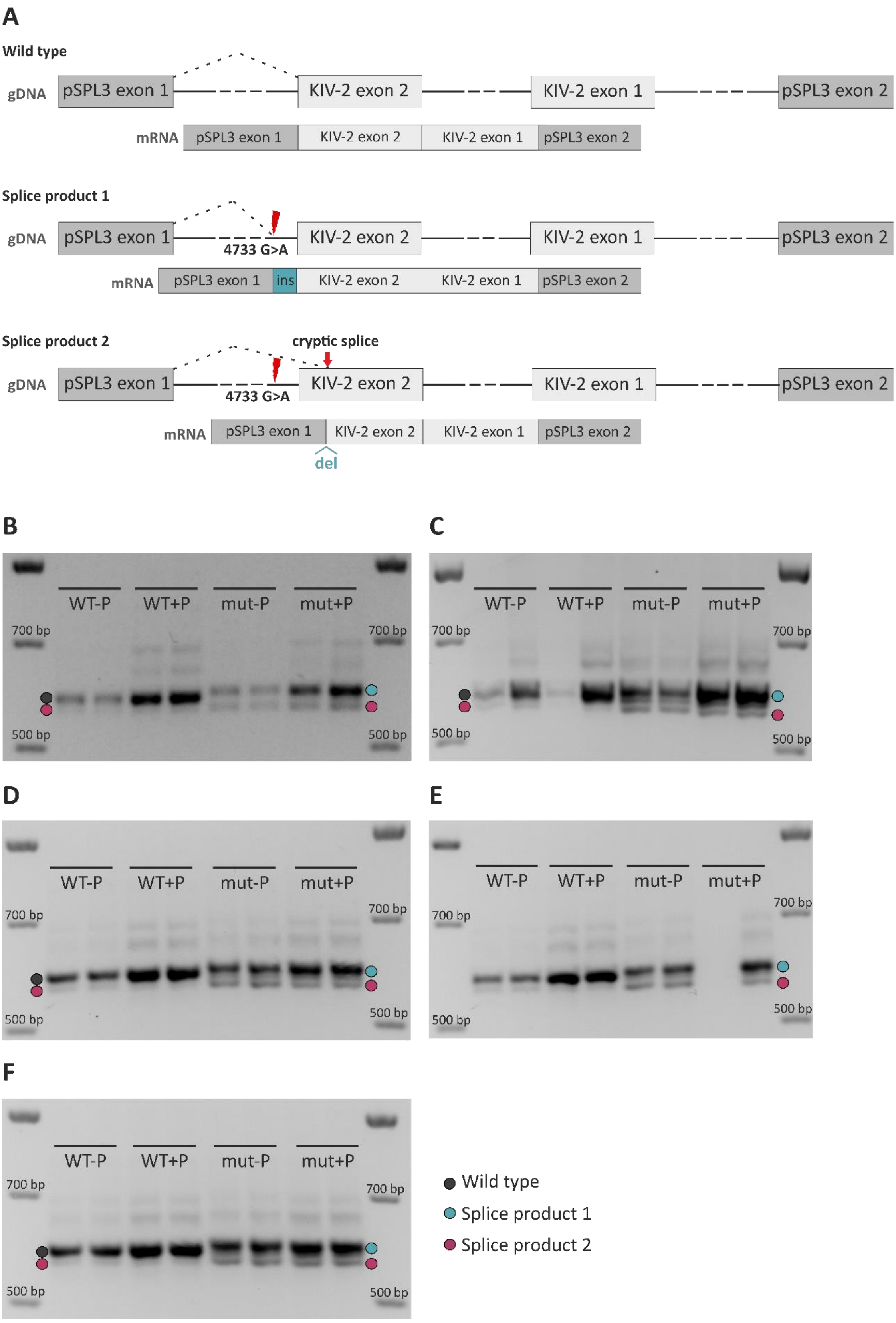
A
Wild type
gDNA
pSPL3 exon 1
KIV-2 exon 2
KIV-2 exon 1
pSPL3 exon 2
mRNA
pSPL3 exon 1
KIV-2 exon 2
KIV-2 exon 1
pSPL3 exon 2
Splice product 1
gDNA
4733 G>A
mRNA
ins
Splice product 2
cryptic splice
gDNA
4733 G>A
mRNA
del
B
C
D
E
F
WT-P
WT+P
mut-P
mut+P
700 bp
500 bp
Wild type
Splice product 1
Splice product 2

Supplemental Figure 7: Minigene assay indicates splicing modulation (shown as Sanger trace).

cDNA Sanger sequence of the mutant minigene PCR product displays a simultaneous 9 bp intron retention and a 24 bp exonic deletion, as deduced from manual separation of the mixed Sanger traces. The explanation of the sequence portions that are mixed are shown below the Sanger trace.

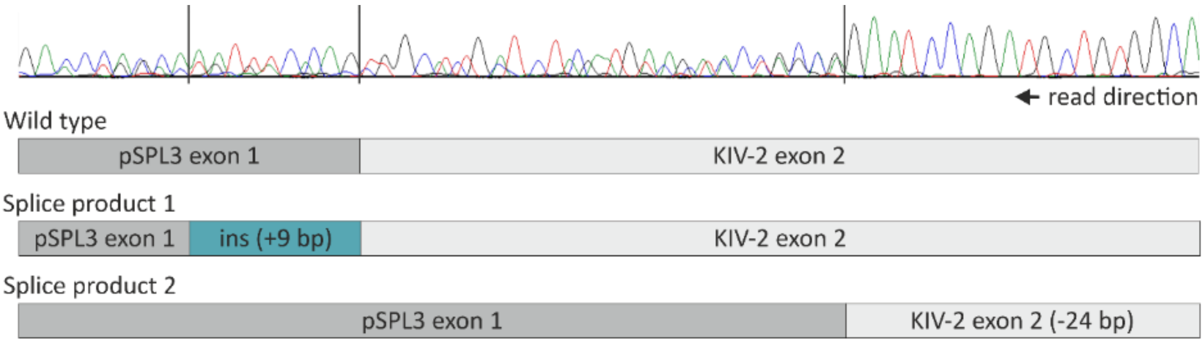

Supplemental Figure 8: Prediction of the effects of 4733G>A on splicing by NetGene2.

Blue: Exon sequence of LPA. Black Intron sequence. Uppercase: position of 4733G>A. Red pipe symbols: predicted acceptor splice sites (pipe symbols denoting the predicted splice event). Green: branchpoint predicted by LaBranchoR, lowercase underlined branchpoint context sequence predicted by SVM-BPfinder (Supplemental methods). SVM-BPfinder predicts the AG exclusion zone to extend for 61 bp upstream of the predict wild type acceptor site (red pipe nr 1).

For the wild type sequence the best scoring splice acceptor coincides with the acceptor splice site of the reference transcript (ENST00000316300, NetGene2 score 0.16; nr 1), with a second weaker splice acceptor predicted downstream (score 0.14; nr 3). For the mutated sequence the wild type splice acceptor is abolished while an additional splice acceptor is created one base downstream of 4733G>A (score 0.25; nr 2). The second predicted splice event downstream of the wild type site is retained (confidence 0.14; nr 3) and a third weak event (score 0.07, unnumbered pipe symbol in the mutated sequence) is predicted even more downstream but was not observed in the minigene products.

Both splice events predicted in the wild type sequence were observed also in the wild type minigenes (see main results). In the mutated minigenes both the event 1 bp downstream of 4733G>A (event nr 2; splice product 1 in Supplemental Figure 6) and the stronger alternative splice event in the exon (event nr 3; splice product 2 in Supplemental Figure 6) were observed.

A. Wild type sequence

```
                                 1                             3
cttgtgacgtgtatcgttttGgaatttccag | tggcttgatcatgaactactgcag | gaatc
cagatgctgtggcagctccttattgttatacgagggatcccggtgtcaggtggga
```

B. Mutated sequence

```
                      2                                        3
cttgtgacgtgtatcgttttAg | aatttccagtggcttgatcatgaactactgcag | gaatc
cag | atgctgtggcagctccttattgttatacgagggatcccggtgtcaggtggga
```

Supplemental Figure 9: Association between Lp(a) concentrations and proxy SNPs in UK Biobank

Lp(a) concentration (nmol/L) in carriers of rs75692336 (purple, proxy SNP for 4925G>A), rs6938647 (orange, proxy SNP for 4733G>A), both variants (green, compound heterozygosity) and none of the variants (blue) in UK Biobank (n=433,563). Both proxy SNPs associate with lower Lp(a) concentrations; in the double carrier group, the variance is reduced. IQR: interquartile range.

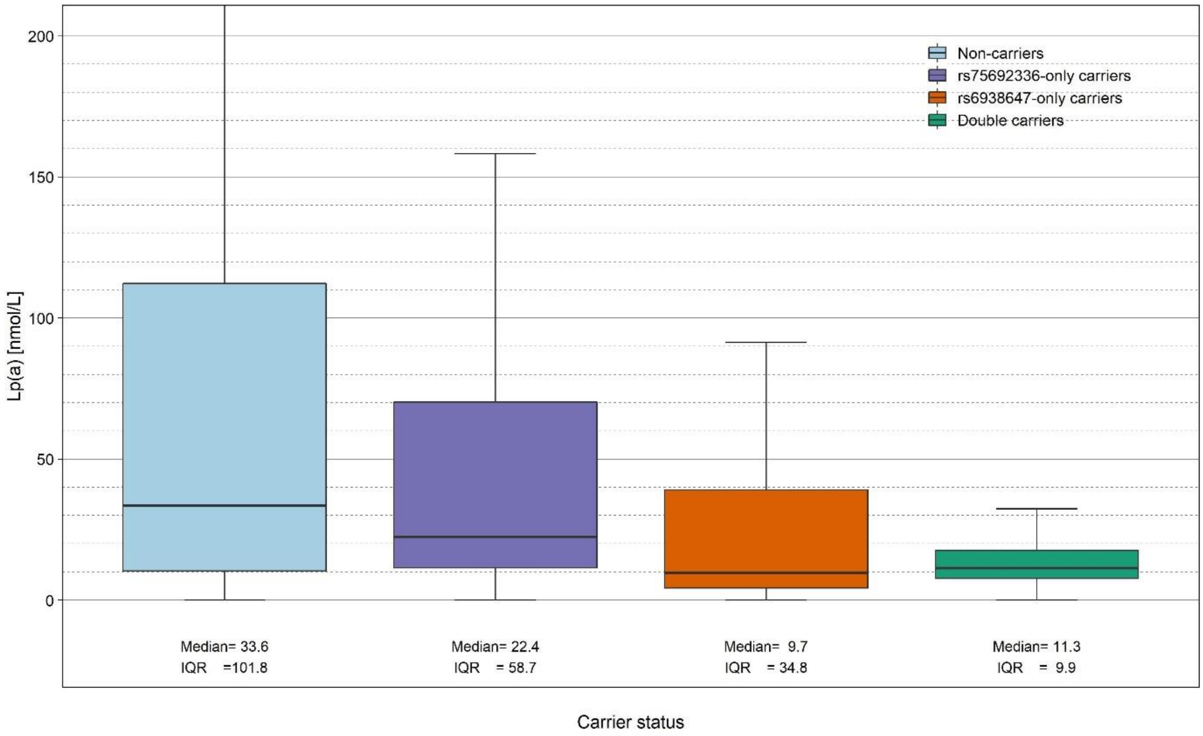

Supplemental Figure 10: Hazard ratio for CAD risk in UK Biobank with time-on-study.

Status refers to the presence (yes) or absence (no) of the proxy SNPs rs6938647/rs75692336 for 4733G>A/4925G>A for the two variants. Reference group are non-carriers of proxy SNPs of both variants. Model is adjusted for sex and age. Time-on-study is taken as time scale. In this plot, individuals with CAD events prior to enrolment are omitted (n=17,835).

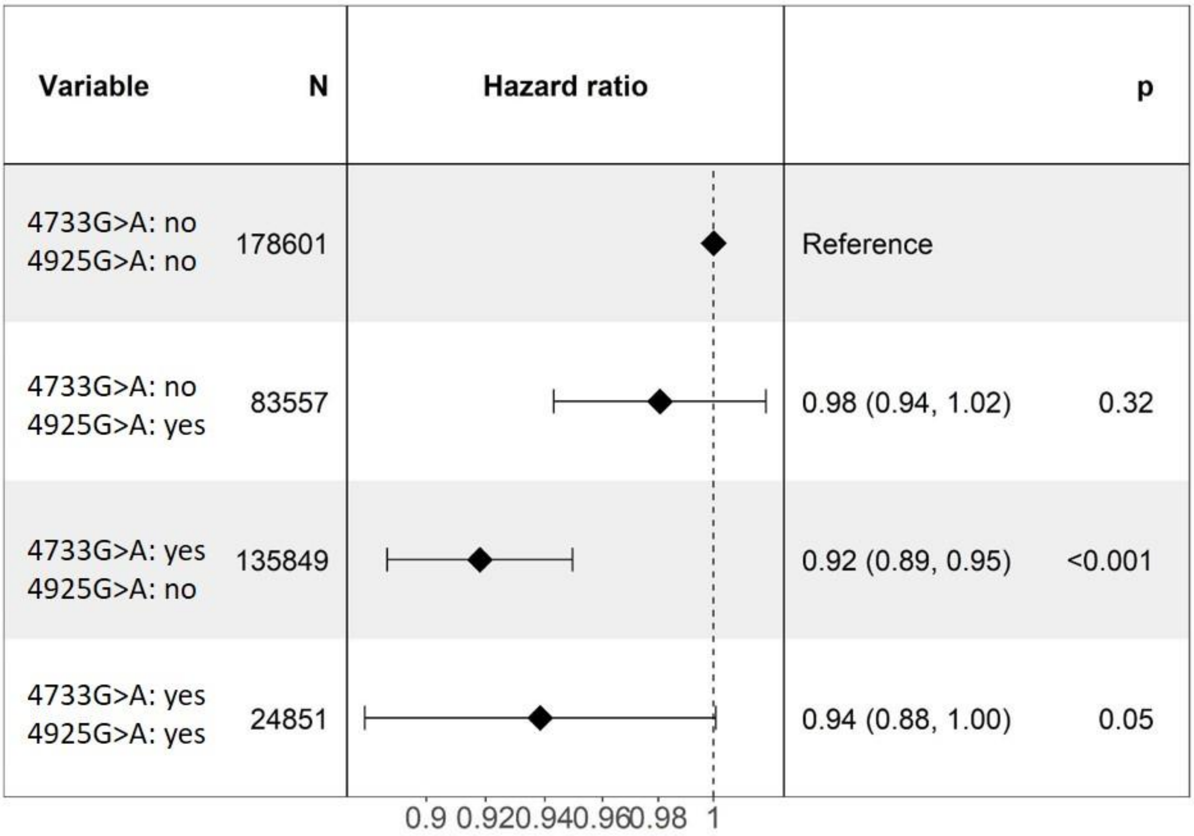

Supplemental Tables

Supplemental Table 1: Positions of 4733G>A in human reference genome hg38 (contains six KIV-2 repeats)

Number of KIV-2 repeat in the human reference genome	Position
1	chr6:160,640,859
2	chr6:160,635,315
3	chr6:160,629,766
4	chr6:160,624,222
5	chr6:160,618,676
6	chr6:160,613,124

Supplemental Table 2: Reaction conditions for 4733G>A asPCR and PNPLA3 control reaction

**Target**	**asPCR *LPA* 4733G>A**	***PNPLA3***
Product length [bp]	160	130
Forward primer	CACCTCTTGTGACGTGTATCGTTTCA*	AATGCCTCCCGGCCAAT
Reverse primer	AGTCCCTTCTGCGTCTGAGCA	CTGCTTACATCCACGACTTCGT
PCR Master Mix	SYBR Select 2x Master Mix	SYBR Select 2x Master Mix
Reaction volume [µL]	5.0	5.0
Final primer concentration [µM each]	0.9	0.9
DNA input [ng]	20.0	20.0
**Cycling conditions**		
Initial denaturation	95 °C, 2 min	
Denaturation	95 °C, 15 sec	
Annealing	64 °C, 15 sec	
Extension	72 °C 1 min	
No. of cycles	40	

* Selective 4733G>A base is shown in red. The additional mismatch at position -2 from the 3'end to enhance the thermodynamic disadvantage of unspecific parings is marked in green.

Supplemental Table 3: Baseline characteristics of the GCKD study

	**GCKD (n=4,673)**
Age (in years)	60.1 ± 11.9 [53.0, 63.0, 70.0]
Women, n (%)	1,862 (39.8%)
Lp(a) (mg/dL), all subjects	24.7 ± 30.6 [5.0, 11.6, 33.8]
LMW isoforms, n (%)	1,151 (24.6%)
LDL cholesterol (mg/dL)	118.4 ± 43.7 [89.2, 113.7, 142.7]
HDL cholesterol (mg/dL)	51.7 ± 17.9 [39.2, 48.2, 61.2]
Total cholesterol (mg/dL)	211.1 ± 52.7 [176.5, 207.0, 239.2]
Triglycerides (mg/dL)	198.1 ± 122.7 [117.5, 168.2, 238.5]
Lipid-lowering medication, n (%)	2,379 (50.9%)
Type 2 Diabetes, n (%)	1,672 (35.8%)
eGFR (mL/min/1.73 m^2)	49.5 ± 18.3 [37.0, 46.0, 58.0]
UACR (mg/g)	466.6 ± 2,949.4 [9.4, 50.5, 373.8]
rs10455872 (MAF)*	0.0636
rs3798220 (MAF)*	0.0176
rs41272114 (MAF)*	0.0258

Continuous variables are shown as mean ± SD and [25%,50%,75%] percentiles

n: Sample size of analysis dataset with available Lp(a)-values (and typing of 4733G>A & 4925G>A)

* Genotypes were available for 4,578 individuals

Supplemental Table 4: Quantile regression analysis for 4925G>A carrier status and Lp(a) levels in the GCKD study.

Model # and adjustment	β [95%CI]	p-value	r^2 by model / 4925G>A *
1: Age, sex, eGFR and isoform 1	-22.6 [-24.1; -21.0]	<0.0001	0.342 / 0.046
2: As model 1 plus 4925G>A	-21.6 [-23.2; -20.1]	<0.0001	0.461 / 0.069
3: As model 2 plus rs41272114	-21.7 [-23.0;-20.3]	<0.0001	0.480 / 0.070
4: As model 3 plus rs10455872 and rs3798220	-9.9 [-11.1; -8.6]	<0.0001	0.511 / 0.0

* Variance explained (r^2) derived from linear model on inverse normal transformed Lp(a) concentrations.

Supplemental Table 5: Relative contribution of the single variables to the fully adjusted linear regression model in Table 1 ordered by magnitude

Regressor	r^2 [95%CIs] of the regressor (pmvd metric of *relaimpo* package in R)
Isoform 1	0.2766 [0.2517;0.2992]
4733G>A	0.1010 [0.0878;0.1152]
4925G>A	0.0453 [0.0377;0.0536]
rs10455872	0.0420 [0.0281;0.0593]
rs3798220	0.0211 [0.0124;0.0321]
rs41272114	0.0201 [0.0136;0.0284]
Sex	0.0041 [0.0019;0.0071]
eGFR	0.0016 [0.0004;0.0039]
Age	0.0003 [0.0000;0.0013]

Supplemental Table 6: Number of individuals and proportions per isoform 1 stratum in Figure 1.

	Isoform 1 stratum [KIV repeats]; Individuals per group																						
	**≤15**	**16/17**	**18/19**	**20**	**21**	**22**	**23**	**24**	**25**	**26**	**27**	**28**	**29**	**30**	**31**	**32**	**33**	**34**	**35**	**36**	**37**	**38**	**≥39**
Non-carriers (n)	48	66	147	256	227	127	220	224	114	103	67	102	110	131	116	139	94	98	103	110	95	73	103
Carriers (n)	20	21	39	70	82	48	93	149	106	113	134	171	132	127	111	86	61	44	41	39	32	32	28

	Isoform 1 stratum [KIV repeats]; Proportions within the stratum in %																						
	**≤15**	**16/17**	**18/19**	**20**	**21**	**22**	**23**	**24**	**25**	**26**	**27**	**28**	**29**	**30**	**31**	**32**	**33**	**34**	**35**	**36**	**37**	**38**	**≥39**
Non-carriers (%)	71	76	79	79	74	73	70	60	52	48	33	37	46	51	51	62	61	69	72	74	75	70	79
Carriers (%)	29	24	21	21	26	27	30	40	48	52	67	62	54	49	48	38	39	31	28	26	25	30	21

Supplemental Table 7: Prediction of the effects of 4733G>A on splicing by Genomnis HSF.

Field	Info
Signal interpretation	Broken WT Acceptor Site: Alteration of the WT Acceptor site, most probably affecting splicing
Prediction tool name	MaxEntScan
Transcript input for prediction	ENST00000316300
Position input for prediction	c.2262-11G>A
Sequence variation considered	TATCGTTTTGGAATTTCCAGTGG > TATCGTTTTAGAATTTCCAGTGG
MaxEnt Score Result	5.25 changed to -0.38 (-107.24%)

Supplemental Table 8: Individuals per carrier group in Figure 3. Proportions (%) within each isoform stratum are given in brackets.

	Isoform 1 stratum [KIV repeats]								
	**≤19**	**20**	**21**	**22**	**23**	**24**	**25**	**26**	**≥27**
non-carriers	203 (59.5)	178 (54.6)	145 (46.9)	85 (48.6)	48 (15.3)	53 (14.2)	58 (26.4)	82 (38.0)	1309 (54.5)
4925G>A only	58 (17.0)	78 (23.9)	82 (26.5)	42 (24.0)	172 (55.0)	171 (45.8)	56 (25.5)	21 (9.7)	44 (1.8)
4733G>A only	66 (19.4)	54 (16.6)	72 (23.3)	33 (18.9)	45 (14.4)	76 (20.4)	83 (37.7)	106 (49.1)	1037 (43.2)
double carriers	14 (4.1)	16 (4.9)	10 (3.2)	15 (8.6)	48 (15.3)	73 (19.6)	23 (10.5)	7 (3.2)	10 (0.4)

Supplemental Table 9: Linkage disequilibrium values for the 10 best proxy SNPs (sorted by r^2). Values from GCKD.

#	SNP	p-value Chi Square	D'	r^2	MAF
1	rs6938647	<0.0001	0.931	0.682	0.232
2	rs6903649	<0.0001	0.953	0.495	0.303
3	rs7770628	<0.0001	0.994	0.260	0.474
4	rs9458010	<0.0001	0.901	0.240	0.445
5	rs4252109	<0.0001	0.661	0.246	0.296
6	rs59614420	<0.0001	0.659	0.240	0.301
7	rs78439586	<0.0001	0.742	0.192	0.077
8	rs1018234	<0.0001	0.481	0.130	0.297
9	rs2457574	<0.0001	0.571	0.098	0.442
10	rs9365169	<0.0001	0.612	0.085	0.488

Supplemental Table 10: Tagging efficiency for the three best proxy SNPs. The three best proxy SNPs for 4733G>A were selected based on the r^2. Other selection criteria which were considered were: High D' and similar MAF compared to the 4733G>A variant. Finally, rs6938647 was chosen as proxy SNP for 4733G>A since it has the highest specificity. The r^2 and D' values for rs75692336 and 4925G>A are taken from reference (1). Genotypes for proxy SNPs were available for 4,578 individuals.

Proxy SNP (tagged variant)	Proxy SNP carriers [n]	Correctly assigned 4733 G>A	Wrongly assigned 4733 G>A	Missed 4733 G>A	Correctly assigned wild type individuals	D'	r^2
rs6938647 (4733G>A)	1,880	1,669	211	91	2607	0.93	0.68
rs6903649 (4733G>A)	2,363	1,708	655	52	2163	0.95	0.495
rs7770628 (4733G>A)	3,312	1,756	1,556	4	1262	0.99	0.26
rs75692336 (4925G>A; for comparison)*	1,039	925	114	1	3538	0.99	0.82

*proxy SNP for 4925G>A from reference (1)

Supplemental Table 11: Frequency of *LPA* KIV-2 4733G>A in the continental groups (super populations) of the 1000 Genomes project. The data is reported without coverage filtering at the variant position and with coverage filter set at 780X (high confidence variant calls according to (3)). The codes of the super populations are explained in Supplemental Table 12.

No coverage filter					
Super population	**AFR**	**AMR**	**EAS**	**EUR**	**SAS**
Carriers [n]	9	72	0	121	26
Total [n]	661	347	504	503	489
Frequency [%]	1.4	20.7	0.0	24.1	5.3
Coverage >780X					
Super population	**AFR**	**AMR**	**EAS**	**EUR**	**SAS**
Carriers [n]	8	66	0	67	24
Total [n]	522	251	152	191	459
Frequency [%]	1.5	26.3	0.0	35.1	5.2

Supplemental Table 12: Frequency of LPA KIV-2 4733G>A in populations of the 1000 Genomes project

		No coverage filter			Coverage >780X		
Population	**Description**	**carriers [n]**	**total [n]**	**frequency [%]**	**carriers [n]**	**total [n]**	**frequency [%]**
Africans (AFR)							
ACB	African Caribbeans in Barbados	2	96	2.1	2	60	3.3
ASW	Americans of African Ancestry in SW USA	7	61	11.5	6	52	11.5
ESN	Esan in Nigeria	0	99	0.0	0	99	0.0
GWD	Gambian in Western Divisions in the Gambia	0	113	0.0	0	113	0.0
LWK	Luhya in Webuye, Kenya	0	99	0.0	0	38	0.0
MSL	Mende in Sierra Leone	0	85	0.0	0	85	0.0
YRI	Yoruba in Ibadan, Nigeria	0	108	0.0	0	75	0.0
Admixed Americans (AMR)							
CLM	Colombians from Medellin, Colombia	18	94	19.1	15	65	23.1
MXL	Mexican Ancestry from Los Angeles USA	15	64	23.4	15	56	26.8
PEL	Peruvians from Lima, Peru	6	85	7.1	5	47	10.6
PUR	Puerto Ricans from Puerto Rico	33	104	31.7	31	83	37.3
East Asians (EAS)							
CDX	Chinese Dai in Xishuangbanna, China	0	93	0.0	0	5	0.0
CHB	Han Chinese in Beijing, China	0	103	0.0	0	21	0.0
CHS	Southern Han Chinese	0	105	0.0	0	22	0.0
JPT	Japanese in Tokyo, Japan	0	104	0.0	0	69	0.0
KHV	Kinh in Ho Chi Minh City, Vietnam	0	99	0.0	0	35	0.0
Europeans (EUR)							
CEU	Utah Residents (CEPH) with Northern and Western European Ancestry	22	99	22.2	12	44	27.3
FIN	Finnish in Finland	16	99	16.2	10	30	33.3
GBR	British in England and Scotland	26	91	28.6	13	35	37.1
IBS	Iberian Population in Spain	25	107	23.4	11	31	35.5
TSI	Toscani in Italia	32	107	29.9	21	51	41.2
South Asian (SAS)							
BEB	Bengali from Bangladesh	3	86	3.5	3	85	3.5
GIH	Gujarati Indian from Houston, Texas	5	103	4.9	3	74	4.1
ITU	Indian Telugu from the UK	6	102	5.9	6	102	5.9
PJL	Punjabi from Lahore, Pakistan	8	96	8.3	8	96	8.3
STU	Sri Lankan Tamil from the UK	4	102	3.9	4	102	3.9

Supplemental Table 13: Variant level of the 4733G>A in all 1000G samples carrying the 4733G>A without coverage filter. The codes of the populations and super populations are explained in Supplemental Table 12.

Sample	Reference/Variant	Variant level	Total sequencing coverage	Population	Super population
HG02334	G/A	0.0619	7029	ACB	AFR
HG02536	G/A	0.0198	1713	ACB	AFR
NA19707	G/A	0.0264	3066	ASW	AFR
NA19711	G/A	0.0226	2827	ASW	AFR
NA19904	G/A	0.0257	3109	ASW	AFR
NA20278	G/A	0.026	4269	ASW	AFR
NA20296	G/A	0.0228	5477	ASW	AFR
NA20342	G/A	0.0269	6091	ASW	AFR
NA20351	G/A	0.0248	363	ASW	AFR
HG00637	G/A	0.0229	5535	PUR	AMR
HG00638	G/A	0.0531	5729	PUR	AMR
HG00641	G/A	0.0374	11940	PUR	AMR
HG00731	G/A	0.0462	22273	PUR	AMR
HG00732	G/A	0.0189	27204	PUR	AMR
HG00737	G/A	0.0198	10518	PUR	AMR
HG00740	G/A	0.0261	3568	PUR	AMR
HG00743	G/A	0.032	2311	PUR	AMR
HG01047	G/A	0.0173	6026	PUR	AMR
HG01048	G/A	0.0178	10102	PUR	AMR
HG01049	G/A	0.0185	10348	PUR	AMR
HG01058	G/A	0.0209	2206	PUR	AMR
HG01067	G/A	0.0179	7313	PUR	AMR
HG01075	G/A	0.0367	3596	PUR	AMR
HG01079	G/A	0.022	5044	PUR	AMR
HG01095	G/A	0.0213	6000	PUR	AMR
HG01098	G/A	0.026	6046	PUR	AMR
HG01102	G/A	0.0437	5773	PUR	AMR
HG01107	G/A	0.0386	207	PUR	AMR
HG01113	G/A	0.0303	5244	CLM	AMR
HG01119	G/A	0.0199	3912	CLM	AMR
HG01133	G/A	0.033	394	CLM	AMR
HG01168	G/A	0.0168	14149	PUR	AMR
HG01171	G/A	0.0353	170	PUR	AMR
HG01177	G/A	0.0248	6127	PUR	AMR
HG01197	G/A	0.0214	9123	PUR	AMR
HG01257	G/A	0.0245	3829	CLM	AMR
HG01259	G/A	0.0371	3743	CLM	AMR
HG01303	G/A	0.025	2237	PUR	AMR
HG01305	G/A	0.0213	1549	PUR	AMR
HG01311	G/A	0.0255	1963	PUR	AMR
HG01312	G/A	0.0215	2144	PUR	AMR
HG01323	G/A	0.0739	1475	PUR	AMR
HG01326	G/A	0.0204	2592	PUR	AMR
HG01344	G/A	0.0247	486	CLM	AMR
HG01357	G/A	0.0286	420	CLM	AMR
HG01372	G/A	0.0231	2939	CLM	AMR
HG01377	G/A	0.0293	4367	CLM	AMR
HG01384	G/A	0.0247	2593	CLM	AMR

… to be continued

Supplemental Table 13: continued

Sample	Reference/Variant	Variant level	Total coverage	Population	Superpopulation
HG01392	G/A	0.0211	3645	PUR	AMR
HG01396	G/A	0.0425	3745	PUR	AMR
HG01403	G/A	0.0284	2886	PUR	AMR
HG01405	G/A	0.0185	4320	PUR	AMR
HG01447	G/A	0.0322	2361	CLM	AMR
HG01459	G/A	0.0218	2390	CLM	AMR
HG01465	G/A	0.0216	3788	CLM	AMR
HG01468	G/A	0.0173	2723	CLM	AMR
HG01474	G/A	0.0144	1878	CLM	AMR
HG01485	G/A	0.0294	2989	CLM	AMR
HG01491	G/A	0.0223	3543	CLM	AMR
HG01492	G/A	0.0212	4248	CLM	AMR
HG01566	G/A	0.032	438	PEL	AMR
HG01967	G/A	0.0288	18015	PEL	AMR
HG01974	G/A	0.0374	29558	PEL	AMR
HG02150	G/A	0.0212	3496	PEL	AMR
HG02253	G/A	0.0202	4203	PEL	AMR
HG02298	G/A	0.0237	5873	PEL	AMR
NA19648	G/A	0.0194	8962	MXL	AMR
NA19654	G/A	0.0211	3750	MXL	AMR
NA19657	G/A	0.016	3382	MXL	AMR
NA19658	G/A	0.0267	2623	MXL	AMR
NA19669	G/A	0.0243	3338	MXL	AMR
NA19670	G/A	0.0194	3756	MXL	AMR
NA19676	G/A	0.0175	3377	MXL	AMR
NA19719	G/A	0.0202	3466	MXL	AMR
NA19722	G/A	0.0548	2904	MXL	AMR
NA19747	G/A	0.0316	11671	MXL	AMR
NA19749	G/A	0.0248	5085	MXL	AMR
NA19770	G/A	0.0218	4683	MXL	AMR
NA19777	G/A	0.0252	4797	MXL	AMR
NA19780	G/A	0.029	4590	MXL	AMR
NA19786	G/A	0.0469	6141	MXL	AMR
HG00096	G/A	0.0264	5494	GBR	EUR
HG00101	G/A	0.0399	4434	GBR	EUR
HG00105	G/A	0.026	346	GBR	EUR
HG00106	G/A	0.0648	293	GBR	EUR
HG00116	G/A	0.0328	3967	GBR	EUR
HG00117	G/A	0.0225	4893	GBR	EUR
HG00120	G/A	0.0171	5833	GBR	EUR
HG00122	G/A	0.0423	355	GBR	EUR
HG00127	G/A	0.0273	2968	GBR	EUR
HG00129	G/A	0.0726	358	GBR	EUR
HG00132	G/A	0.0242	579	GBR	EUR
HG00137	G/A	0.0462	130	GBR	EUR
HG00141	G/A	0.0291	481	GBR	EUR
HG00142	G/A	0.0313	479	GBR	EUR
HG00146	G/A	0.021	8798	GBR	EUR
HG00149	G/A	0.0538	316	GBR	EUR
HG00150	G/A	0.0263	418	GBR	EUR
HG00157	G/A	0.0171	4333	GBR	EUR
HG00160	G/A	0.0385	4314	GBR	EUR

… to be continued

Supplemental Table 13: continued

Sample	Reference/Variant	Variant level	Total coverage	Population	Superpopulation
HG00181	G/A	0.0276	5511	FIN	EUR
HG00187	G/A	0.0232	6891	FIN	EUR
HG00188	G/A	0.0416	7443	FIN	EUR
HG00231	G/A	0.02	500	GBR	EUR
HG00233	G/A	0.056	5783	GBR	EUR
HG00235	G/A	0.0412	267	GBR	EUR
HG00240	G/A	0.0245	5263	GBR	EUR
HG00242	G/A	0.0217	6594	GBR	EUR
HG00262	G/A	0.0209	11649	GBR	EUR
HG00266	G/A	0.0277	361	FIN	EUR
HG00268	G/A	0.0426	188	FIN	EUR
HG00304	G/A	0.0244	1268	FIN	EUR
HG00308	G/A	0.0263	8291	FIN	EUR
HG00310	G/A	0.0484	310	FIN	EUR
HG00311	G/A	0.0198	3437	FIN	EUR
HG00351	G/A	0.0362	359	FIN	EUR
HG00355	G/A	0.0475	316	FIN	EUR
HG00357	G/A	0.0211	3552	FIN	EUR
HG00361	G/A	0.0238	5974	FIN	EUR
HG00364	G/A	0.0216	603	FIN	EUR
HG00367	G/A	0.0596	2364	FIN	EUR
HG00379	G/A	0.0325	1999	FIN	EUR
HG01521	G/A	0.0298	11609	IBS	EUR
HG01522	G/A	0.0221	6593	IBS	EUR
HG01606	G/A	0.0484	351	IBS	EUR
HG01608	G/A	0.0561	303	IBS	EUR
HG01619	G/A	0.0372	538	IBS	EUR
HG01620	G/A	0.0304	395	IBS	EUR
HG01624	G/A	0.0149	606	IBS	EUR
HG01626	G/A	0.0516	310	IBS	EUR
HG01628	G/A	0.0195	461	IBS	EUR
HG01632	G/A	0.0361	305	IBS	EUR
HG01684	G/A	0.0424	165	IBS	EUR
HG01699	G/A	0.052	7685	IBS	EUR
HG01704	G/A	0.0275	8632	IBS	EUR
HG01762	G/A	0.0316	14543	IBS	EUR
HG01767	G/A	0.0259	2777	IBS	EUR
HG01773	G/A	0.027	592	IBS	EUR
HG01777	G/A	0.0387	336	IBS	EUR
HG01784	G/A	0.0551	345	IBS	EUR
HG01785	G/A	0.0365	1152	IBS	EUR
HG01786	G/A	0.029	1276	IBS	EUR
HG01789	G/A	0.0403	447	GBR	EUR
HG02219	G/A	0.0197	2280	IBS	EUR
HG02220	G/A	0.0253	277	IBS	EUR
HG02223	G/A	0.0181	10203	IBS	EUR
HG02231	G/A	0.0313	6317	IBS	EUR
HG02235	G/A	0.0436	436	IBS	EUR
NA07051	G/A	0.0456	4385	CEU	EUR
NA07056	G/A	0.0458	349	CEU	EUR

… to be continued

Supplemental Table 13: continued

Sample	Reference/Variant	Variant level	Total coverage	Population	Superpopulation
NA10851	G/A	0.0398	327	CEU	EUR
NA11843	G/A	0.025	3764	CEU	EUR
NA11918	G/A	0.0217	11849	CEU	EUR
NA11919	G/A	0.0225	4271	CEU	EUR
NA11920	G/A	0.0225	7927	CEU	EUR
NA11932	G/A	0.0268	224	CEU	EUR
NA11994	G/A	0.0416	433	CEU	EUR
NA12005	G/A	0.0321	499	CEU	EUR
NA12006	G/A	0.0226	399	CEU	EUR
NA12287	G/A	0.0287	174	CEU	EUR
NA12340	G/A	0.019	3481	CEU	EUR
NA12750	G/A	0.0203	7156	CEU	EUR
NA12751	G/A	0.0197	6287	CEU	EUR
NA12763	G/A	0.0281	4513	CEU	EUR
NA12777	G/A	0.0543	184	CEU	EUR
NA12812	G/A	0.0385	312	CEU	EUR
NA12830	G/A	0.0169	3965	CEU	EUR
NA12872	G/A	0.0308	325	CEU	EUR
NA12878	G/A	0.019	4991	CEU	EUR
NA12890	G/A	0.0296	6318	CEU	EUR
NA20503	G/A	0.0286	419	TSI	EUR
NA20510	G/A	0.02	600	TSI	EUR
NA20511	G/A	0.0251	2309	TSI	EUR
NA20512	G/A	0.0454	551	TSI	EUR
NA20514	G/A	0.0106	565	TSI	EUR
NA20515	G/A	0.0222	360	TSI	EUR
NA20518	G/A	0.0457	328	TSI	EUR
NA20525	G/A	0.0272	367	TSI	EUR
NA20528	G/A	0.0293	547	TSI	EUR
NA20533	G/A	0.0414	338	TSI	EUR
NA20534	G/A	0.0489	409	TSI	EUR
NA20538	G/A	0.0343	496	TSI	EUR
NA20587	G/A	0.0426	6147	TSI	EUR
NA20764	G/A	0.0202	3763	TSI	EUR
NA20767	G/A	0.0196	4390	TSI	EUR
NA20769	G/A	0.0228	3768	TSI	EUR
NA20787	G/A	0.0204	4109	TSI	EUR
NA20796	G/A	0.0192	5582	TSI	EUR
NA20797	G/A	0.0236	6682	TSI	EUR
NA20804	G/A	0.0256	3981	TSI	EUR
NA20808	G/A	0.0285	4138	TSI	EUR
NA20809	G/A	0.0266	4919	TSI	EUR
NA20812	G/A	0.0243	6796	TSI	EUR
NA20813	G/A	0.0276	5146	TSI	EUR
NA20815	G/A	0.0218	11563	TSI	EUR
NA20818	G/A	0.0246	10182	TSI	EUR
NA20821	G/A	0.0249	2970	TSI	EUR
NA20822	G/A	0.0217	3045	TSI	EUR
NA20826	G/A	0.0182	8979	TSI	EUR
NA20827	G/A	0.0301	1096	TSI	EUR

… to be continued

Supplemental Table 13: continued

Sample	Reference/Variant	Variant level	Total coverage	Population	Superpopulation
NA20828	G/A	0.0187	9123	TSI	EUR
NA20832	G/A	0.0269	1638	TSI	EUR
HG01586	G/A	0.0182	1539	PJL	SAS
HG02600	G/A	0.0231	7689	PJL	SAS
HG02651	G/A	0.0204	3877	PJL	SAS
HG02725	G/A	0.0194	10639	PJL	SAS
HG02780	G/A	0.0282	1242	PJL	SAS
HG03022	G/A	0.0425	1648	PJL	SAS
HG03488	G/A	0.0176	6419	PJL	SAS
HG03490	G/A	0.0145	1793	PJL	SAS
HG03698	G/A	0.0198	2984	STU	SAS
HG03731	G/A	0.0293	4610	ITU	SAS
HG03789	G/A	0.0208	4751	ITU	SAS
HG03922	G/A	0.0217	2851	BEB	SAS
HG03931	G/A	0.0203	4046	BEB	SAS
HG03945	G/A	0.0252	3289	STU	SAS
HG03960	G/A	0.0186	7490	ITU	SAS
HG03990	G/A	0.0371	1321	STU	SAS
HG04002	G/A	0.021	3184	ITU	SAS
HG04020	G/A	0.0269	7429	ITU	SAS
HG04038	G/A	0.0162	1295	STU	SAS
HG04188	G/A	0.0185	3021	BEB	SAS
HG04219	G/A	0.0221	1809	ITU	SAS
NA20858	G/A	0.0251	9350	GIH	SAS
NA20864	G/A	0.0352	227	GIH	SAS
NA20886	G/A	0.0231	9679	GIH	SAS
NA20905	G/A	0.0261	9304	GIH	SAS
NA21107	G/A	0.0199	452	GIH	SAS

Supplemental Table 14: PCR for site directed mutagenesis.

**Product length [bp]**	10422
**Enzyme**	KAPA HiFi DNA Polymerase
**Forward primer (base for mutagenesis shown in red)**	CTTGTGACGTGTATCGTTTTAGAATTTCCAGTGGCTTGATC
**Reverse primer (base for mutagenesis shown in red)**	GATCAAGCCACTGGAAATTCTAAAACGATACACGTCACAAG
**Reaction volume [µL]**	50
**Enzyme amount [µL]**	1
**Final primer concentration [µM each]**	0.3
**Final dNTP concentration [mM]**	0.3
**DNA input [ng]**	25
**Initial denaturation**	95 °C, 3 min
**Denaturation**	98 °C, 20 sec
**Annealing**	67.5 °C, 15 sec
**Extension**	72 °C 5 min
**Final extension**	72 °C 10 min
**No. of cycles**	15

Supplemental Methods

Variant naming

Genomic localization of variants in the KIV-2 to the genome is ambiguous and thus no rs identifier can be attributed. As for 4925G>A we therefore name the variant 4733G>A according to its position in the reference sequence used for the NGS mutation screening (1). Possible genome coordinates are given in Supplemental Table 1. The base change is given relative to the coding strand of LPA NM_005577.4.

Assay development

Several allele-specific primers were designed containing the selective allele at the 3' end. Additional thermodynamic disadvantage to unspecific pairings is given by various base mismatches introduced on position -2 from the 3' end of the allele-specific primers (4). No variants in the primer binding sites were observed in the European populations from the 1000G project (data from (3)). Performance of these primers was tested in mixtures of pSPL3 plasmids containing 4733 wild type or mutant and the primer with the highest specificity and sensitivity was chosen. Primer concentrations were optimized using one sample containing the 4733G>A variant and one without, as confirmed by ultra-deep NGS (3). The final protocol was again tested for sensitivity on mixtures of pSPL3 plasmids containing 4733 wild type or mutant ranging from 100% to 0% mutant fraction (Supplemental Figure 2). In addition, we used also 16 samples from SAPHIR (5) (8 which are confirmed non-carriers of the variant and 8 with variant levels ranging from 1.7 to 6.4 % variant level determined by ultra-deep NGS data from Coassin, Schönherr et al., 2019 (3)) to further validate the assay and determine the optimal C_T threshold. A PCR amplicon for *PNPLA3* served as positive amplification control to detect PCR failures e.g. due to insufficient DNA quality. PCR reactions were run on an Applied Biosystems QuantStudio 6 Flex system in 384 well format using SYBR green chemistry.

Assay validation

The allele-specific real-time PCR (asPCR) assay correctly classified all 59 GCKD samples where 4733G>A carrier status had been confirmed previously by ultra-deep next generation sequencing (3) (23 positives and 36 negative samples). The positive samples showed a mutational level (representing the fraction of mutated KIV-2 units) from 1.86 to 10.04 %, corresponding to Ct values ranging from 29.95 to 33.02. All 39 negative samples showed either no amplification or Ct values >38.3. Thus the separation of the two groups was very clear. A Ct >35 was considered negative in GCKD typing. Of 4,909 GCKD samples typed only two samples had to be excluded due to failure of the amplification control product (indicating e.g. insufficient DNA quality or quantity).

Description of the GCKD study

The GCKD study comprises of Caucasian patients with moderate CKD. Moderate CKD is defined as an estimated glomerular filtration rate (eGFR) according to the CKD_EPI equation of 30-60 mL/min per 1.73 m^2 (corresponding to CKD Stage 3) or overt proteinuria and eGFR > 60 mL/min per 1.73 m^2. Overt proteinuria is defined as an albumin to creatinine ratio in the urine of >300 mg/g or a protein to creatinine ratio in 24-h urine of >500mg/g. Exclusion criteria were active malignancy, NYHA IV heart failure, renal or any other transplantation, non-Caucasian origin and legal attendance. The study has

been approved by the review boards of the participating institutions and informed consent was obtained from all the participants.

ELISA and Western blot in GCKD

For determination of the Lp(a) concentrations by ELISA, plates were coated using a polyclonal affinity-purified rabbit anti-human apo(a) antibody to immobilize the Lp(a) particles. The horseradish-peroxidase-conjugated monoclonal anti-apo(a) antibody 1A2 (6) was used for detection. Each sample was measured twice at 1:150 and 1:1500 dilution and the OD reading, which was in the linear range of the 7-point standard curve, was used. The lower detection limit of the assay is 0.1 mg/dL. A detailed protocol has been published in (6).

For isoform determination 150 ng Lp(a) of each sample were loaded on a 1.46% agarose gel with 0.08% SDS. Electrophoresis time was 18 h at 0.04 A constant current. A size standard containing five plasma samples with only one apo(a) isoform each (13, 19, 23, 27, 35 KIV repeats) was applied in every seventh lane of the gel. Gel was semi-dry blotted to a PVDF membrane blocked with 1% BSA, 85 mM NaCl, 10 mM TRIS, 0.2% Triton X-100 for 30 min at 37°C. The membrane was incubated with horseradish-peroxidase-conjugated 1A2 antibody, washed extensively and signals were detected using ECL substrate (WesternBright Chemilumineszenz Spray, Biozym, Vienna, AT; Amersham Hyperfilm™ ECL™, GE Healthcare, Chicago, IL, USA). A detailed protocol has been published in (6).

Individuals without any isoform expressed but Lp(a) values available are included in the analysis, but excluded from plots showing isoform data.

Look up in public datasets

The three best proxy SNPs for 4733G>A were selected based on the R2. Other selection criteria which were considered were: High D' and similar MAF compared to the 4733G>A variant. The direction of the OR reported in the dataset was adjusted to match the tagging allele. For assessing the coronary artery disease (CAD) risk for carrying both 4733G>A and 4925G>A, rs6938647 was used as proxy SNP for 4733G>A since the combination of rs6938647 (for 47233G>A) and rs75692336 (for 4925G>A) was the proxy SNP combination with the highest specificity (Supplemental Table 10).

Frequency in 1000 Genomes

The frequency of LPA KIV-2 4733G>A in the 1000 Genome (1000G) data was assessed as described previously (1, 3). In brief, the 1000G phase 3 high coverage exome data that mapped to the LPA KIV-2 region (GRCh37, chr6:161,033,785–161,066,618) were downloaded as BAM file from ftp://ftp.1000genomes.ebi.ac.uk/vol1/ftp/phase3/data and submitted to our LPA Server Pipeline (3). Since all reads are aligned to one single repeat, variants present in one or a few KIV-2 repeats are detected as low level mutations, resembling the calling of somatic mutation. A coverage of 780X is required for high confidence calls (i.e. variant calling at ≥1% mutation level with the 95% confidence interval (95%CI) of a binomial distribution not crossing zero) in paired end sequencing data (3).

Survival analysis and Lp(a) concentrations in UK Biobank

The UK Biobank study recruited 503,325 individuals between 2006-2010 (age at enrolment 40-69 years) and was approved by the North West Multi-centre Research Ethics Committee. The Lp(a) concentrations were measured by an immunoturbidimetric assay (Randox Laboratories; Crumlin, County Antrim, United Kingdom using a Beckman Coulter AU5800 Platform). Concentrations falling

within the reportable range of the assay (3.8-189 nmol/L) are available in the UK Biobank showcase. Lp(a) levels outside the reportable range were requested separately. The number of UK Biobank participants with Lp(a) levels below and above the reportable range are 46,836 and 32,953, respectively. Genetic data available from UK Biobank were generated as described elsewhere(7).

We restricted our analyses to Caucasians by selecting participants with British, Irish or any other white ethnic background (Data-Field 21000, n=472,125). First, we investigated in UK Biobank the Lp(a)-lowering effect of the proxy SNPs for 4925G>A (rs75692336) and 4733G>A (rs6938647) as well as their combination (n=433,563 with Lp(a) concentrations available; among them 45,533 (10.5%) and 31,051 (7.16%) individuals had Lp(a) levels below and above reportable range, respectively). Subsequently, we also investigated their protective effect on CAD (n=440,234 with genotyping data available). The incidence of CAD was assessed based on first reported occurrences of diagnoses falling within the codes I21-I25 (8) of the International Classification of Disease version 10 (ICD-10), covering acute myocardial infarction, subsequent myocardial infarction, certain current complications following acute myocardial infarction, other acute ischaemic heart diseases and chronic ischaemic heart diseases (Data-Fields 131298, 121300, 131302, 131304, 131306). The first reported occurrence was provided by mapping the earliest date recorded through either self-report at any assessment centre, inpatient hospital data, primary care or death record data. One individual was excluded due to an implausible date of CAD event. Hazard ratio for CAD was estimated as a function of the carrier status for the proxy SNPs adjusted for sex by cox proportional hazard regression with age as time scale (9), with censoring at the 1st of January 2020 and year of birth as beginning of observation time. In total, 35,863 individuals developed an event. Of those, 17,835 events occurred before the recruitment date. The proportional hazard assumption was verified by visual assessment of Schoenfeld residuals.

Bioinformatic predictions

In-silico analysis of the effects of 4733G>A on splicing was done using NetGene2 (10, 11) (http://www.cbs.dtu.dk/services/NetGene2/), the Human Splice Finder (12) (https://hsf.genomnis.com). NetGene2 and NNSplice predictions were done using a ± 100 bp context sequence of 4733G>A according to hg38. Human Splice Finder settings and result are reported in Supplemental Table 7. AGEZ region and branch point was detected by SVM-BPfinder (http://regulatorygenomics.upf.edu/Software/SVM_BP/) (13) and LSTM Branchpoint Retriever (http://bejerano.stanford.edu/labranchor/) (14).

Minigene assay

We used the previously described LPA-pSPL3 minigene (1). The structure of the minigene is given in Supplemental Figure 1. The 4733G>A mutation was introduced by site-directed PCR mutagenesis (Supplemental Table 14) and the complete *LPA* insert (2.6 kb) was verified by Sanger sequencing.

HepG2 hepatoblastoma cells (ATCC_HB-8065) were cultured in DMEM high glucose, pyruvate (Gibco, UK) supplemented with 10% fetal calf serum and 1% final concentration PenStrep (100 u/mL Penicillin and 0.1 mg/mL Streptomycin; Sigma Aldrich). 4 x10^5 cells/well were seeded in 6-well culture plates 24 hours prior to transfection and were incubated (5%CO_2 at 37°C). The cells were transfected with 2.5 µg plasmid using the Transporter™ 5 Transfection Reagent (Polysciences Europe GmbH) in two technical and five biological replicates according to manufacturer's protocol and incubated with and without puromycin (10 µg/mL final concentration) for 5 hours prior to the harvesting of cells. Transfection success was assessed by fluorescence microscopy and transfection efficiencies examined

using LUNA-FL Dual Florescence Cell Counter (Logos Biosystems). Cells were harvested 48 hours after transfection and RNA extraction was performed using TRI Reagent Solution (Invitrogen). The RNA quality was assessed by automated fragment analysis (Agilent Fragment Analyzer, kit DNF471). The RQN ranges from 7.6 to 10 (mean ± SD: 9.7 ± 0.6). 1 µg of RNA was reverse transcribed using AMV Reverse Transcriptase and random hexamers (Promega) and analyzed by PCR and Sanger sequencing as described(1).

Splicing patterns were assessed by PCR using primers that bind in the constitutive minigene exons that flank the *LPA* insert as described in (1) and Supplemental Figure 1.

References

1. Coassin S, Erhart G, Weissensteiner H, et al. A novel but frequent variant in LPA KIV-2 is associated with a pronounced Lp(a) and cardiovascular risk reduction. Eur. Heart J. 2017;38:1823–1831.

2. Lobentanz EM, Krasznai K, Gruber A, et al. Intracellular metabolism of human apolipoprotein(a) in stably transfected Hep G2 cells. Biochemistry 1998;37:5417–5425.

3. Coassin S, Schönherr S, Weissensteiner H, et al. A comprehensive map of single-base polymorphisms in the hypervariable LPA kringle IV type 2 copy number variation region. J. Lipid Res. 2019;60:186–199.

4. You FM, Huo N, Gu YQ, et al. BatchPrimer3: a high throughput web application for PCR and sequencing primer design. BMC Bioinformatics 2008;9:253.

5. Heid IM, Wagner SA, Gohlke H, et al. Genetic architecture of the APM1 gene and its influence on adiponectin plasma levels and parameters of the metabolic syndrome in 1,727 healthy Caucasians. Diabetes 2006;55:375–384.

6. Erhart G, Lamina C, Lehtimäki T, et al. Genetic Factors Explain a Major Fraction of the 50% Lower Lipoprotein(a) Concentrations in Finns. Arterioscler. Thromb. Vasc. Biol. 2018;38:1230–1241.

7. Bycroft C, Freeman C, Petkova D, et al. The UK Biobank resource with deep phenotyping and genomic data. Nature 2018;562:203–209.

8. van der Harst P, Verweij N. Identification of 64 Novel Genetic Loci Provides an Expanded View on the Genetic Architecture of Coronary Artery Disease. Circ. Res. 2018;122:433–443.

9. Inouye M, Abraham G, Nelson CP, et al. Genomic Risk Prediction of Coronary Artery Disease in 480,000 Adults: Implications for Primary Prevention. J. Am. Coll. Cardiol. 2018;72:1883–1893.

10. Hebsgaard SM, Korning PG, Tolstrup N, Engelbrecht J, Rouzé P, Brunak S. Splice site prediction in Arabidopsis thaliana pre-mRNA by combining local and global sequence information. Nucleic Acids Res. 1996.

11. Brunak S, Engelbrecht J, Knudsen S. Prediction of human mRNA donor and acceptor sites from the DNA sequence. J. Mol. Biol. 1991;220:49–65.

12. Desmet FO, Hamroun D, Lalande M, Collod-Beroud G, Claustres M, Beroud C. Human Splicing Finder: an online bioinformatics tool to predict splicing signals. Nucleic Acids Res 2009;37:e67.

13. Corvelo A, Hallegger M, Smith CWJ, Eyras E. Genome-wide association between branch point properties and alternative splicing. PLoS Comput. Biol. 2010;6:e1001016.

14. Paggi JM, Bejerano G. A sequence-based, deep learning model accurately predicts RNA splicing branchpoints. RNA 2018;24:1647–1658.
